# KDM8/JMJD5 as a dual coactivator of AR and PKM2 integrates AR/EZH2 network and tumor metabolism in CRPC

**DOI:** 10.1038/s41388-018-0414-x

**Published:** 2018-08-02

**Authors:** Hung-Jung Wang, Mamata Pochampalli, Ling-Yu Wang, June X Zou, Pei-Shan Li, Sheng-Chieh Hsu, Bi-Juan Wang, Shih-Han Huang, Ping Yang, Joy C. Yang, Cheng-Ying Chu, Chia-Ling Hsieh, Shian-Ying Sung, Chien-Feng Li, Clifford G. Tepper, David K. Ann, Allen C. Gao, Christopher P. Evans, Yoshihiro Izumiya, Chi-Pin Chuu, Wen-Ching Wang, Hong-Wu Chen, Hsing-Jien Kung

**Affiliations:** 1 Institute of Biotechnology and Pharmaceutical Research, National Health Research Institutes, 35053 Miaoli County, Taiwan; 2 Institute of Molecular and Genomic Medicine, National Health Research Institutes, 35053 Miaoli County, Taiwan; 3 Department of Biochemistry and Molecular Medicine, School of Medicine, University of California, Davis, CA 95817 USA; 4 Institute of Biotechnology, National Tsing-Hua University, 30035 Hsinchu, Taiwan; 5 Institute of Cellular and System Medicine, National Health Research Institutes, 35053 Miaoli County, Taiwan; 6 Department of Urology, School of Medicine, University of California, Davis, CA 95817 USA; 7 State Key Laboratory of Oncology in South China, Collaborative Innovation Center for Cancer Medicine, Sun Yat-sen University Cancer Center, Guangzhou, China; 8 Ph.D. Program for Translational Medicine, College of Medical Science and Technology, Taipei Medical University, Taipei City, Taiwan; 9 National Institute of Cancer Research, National Health Research Institutes, 35053 Miaoli County, Taiwan; 10 Department of Molecular Pharmacology, Beckman Research Institute, City of Hope, Duarte, CA USA; 11 Comprehensive Cancer Center, School of Medicine, University of California, Davis, Sacramento, CA USA; 12 Institute of Molecular and Cellular Biology, National Tsing-Hua University, Hsinchu, Taiwan

**Keywords:** Prostate cancer, Cancer metabolism

## Abstract

During the evolution into castration or therapy resistance, prostate cancer cells reprogram the androgen responses to cope with the diminishing level of androgens, and undergo metabolic adaption to the nutritionally deprived and hypoxia conditions. AR (androgen receptor) and PKM2 (pyruvate kinase M2) have key roles in these processes. We report in this study, KDM8/JMJD5, a histone lysine demethylase/dioxygnase, exhibits a novel property as a dual coactivator of AR and PKM2 and as such, it is a potent inducer of castration and therapy resistance. Previously, we showed that KDM8 is involved in the regulation of cell cycle and tumor metabolism in breast cancer cells. Its role in prostate cancer has not been explored. Here, we show that KDM8’s oncogenic properties in prostate cancer come from its direct interaction (1) with AR to affect androgen response and (2) with PKM2 to regulate tumor metabolism. The interaction with AR leads to the elevated expression of androgen response genes in androgen-deprived conditions. They include ANCCA/ATAD2 and EZH2, which are directly targeted by KDM8 and involved in sustaining the survival of the cells under hormone-deprived conditions. Notably, in enzalutamide-resistant cells, the expressions of both KDM8 and EZH2 are further elevated, so are neuroendocrine markers. Consequently, EZH2 inhibitors or KDM8 knockdown both resensitize the cells toward enzalutamide. In the cytosol, KDM8 associates with PKM2, the gatekeeper of pyruvate flux and translocates PKM2 into the nucleus, where the KDM8/PKM2 complex serves as a coactivator of HIF-1α to upregulate glycolytic genes. Using shRNA knockdown, we validate KDM8’s functions as a regulator for both androgen-responsive and metabolic genes. KDM8 thus presents itself as an ideal therapeutic target for metabolic adaptation and castration-resistance of prostate cancer cells.

## Introduction

One of the most troubling aspects of prostate cancer (PCa) progression is the conversion from androgen-dependent to independent (or castration-resistant) state, which at present defies any effective treatment. The next-generation anti-androgens, enzalutamide, and abiraterone have improved the prospects, but eventually therapy-resistant PCa still developed. During the evolution into castration or therapy resistance, the tumor cells need to reprogram the androgen responses to cope with the diminishing level of androgens, and to undergo metabolic adaption to the nutritional and hypoxia conditions during therapy. Increased aerobic glycolysis has been shown to be associated with castration or therapy resistance [1–3]. Nearly all glycolytic genes are overexpressed in advanced PCa [4], many of which are targets of HIF-1α [5]. One of the key factors regulating glycolysis in tumor cells is PKM2, a cytosolic enzyme which controls the level of pyruvate and its flow to lactate versus mitochondria. Through ligand stimulation and post-translational modifications, PKM2 can be translocated into nucleus to become a coactivator for HIF-1α, thereby further fueling the glycolytic pathway [6, 7]. Not surprisingly, overexpression and modifications of PKM2 are associated with PCa progression [8]. In the present study, we describe a novel lysine demethylase which interacts with both AR and PKM2 to reprogram androgen responses and tumor metabolism.

Lysine demethylases (KDMs) have emerged as an important class of epigenetic factors involved in carcinogenesis. Among the eight KDM subfamilies, nearly all (e.g., KDM1, KDM2A-C. KDM3A, C, KDM4A-D, KDM5A-C, KDM6B, C, KDM8 (this study)) have been found to be overexpessed in PCa, and several of them correlate with poor prognosis, indicating a critical role of histone demethylation in tumorigenesis [9]. The most well-characterized are the KDM1 and KDM4 subfamilies. It was found that KDM1, KDM4A, B and C all associate with AR and serve as coactivators [10, 11], and their overexpressions predict poor prognosis of PCa [12, 13]. We recently reported that genetic and pharmacological inhibitors to KDM4A and 4B suppress the growth of PCa cells without affecting normal prostate epithelial cells [14]. Duan et al. [15] reported a similar finding with another KDM4A inhibitor. These data suggest that KDMs are potential targets for PCa therapy.

KDM8/JMJD5, the newest member of the histone demethylase family, is involved in embryogenesis [16], oncogenesis [16, 17], and stem-cell renewal [18]. Overexpression of KDM8 was observed in a variety of tumor tissues [17, 19, 20] and knockdown of KDM8 compromised the growth of cancer cells [16, 17] (and this study). Together, they suggest a critical role of KDM8 in development and cell growth. We and others [16, 17, 21–24] showed that KDM8 is associated with H3K36me2 demethylation in vivo. Macron et. al. identified RCCD1, a centromere and DNA-binding protein, can augment KDM8’s activity [23, 25].Other studies suggest that KDM8 may also function as a dioxygenase [26], and as an aminopeptidase, digesting methylated histone tails to modulate chromatin conformation [27, 28]. Accumulating evidence suggest that KDM8 is a key cell cycle regulator by upregulating Cyclin A [17, 22], modulation of the expressions of p53 and p21 [16, 18, 22, 29, 30] and interactions with spindle microtubules [31]. In addition, it is involved in regulating the fidelity of centrosome duplication by suppression the expression of Satellite repeats [23] and DNA recombination [24]. KDM8 shuttles between cytosol and nucleus with the NLS recently mapped at the N terminus with binding to importin [32]. Interestingly, we reported that KDM8 facilitates the nuclear translocation of PKM2 (pyruvate kinase M2 isoform), a critical enzyme involved in tumor metabolism [7]. We found that KDM8 binds PKM2 and enhances the conversion of cytosolic tetramer form of PKM2 into the dimer or heterodimer form which enters nucleus. Together with KDM8, PKM2 serves as a coactivator of HIF-1α, to upregulate enzymes involved in Warburg effect [7]. All data, taken together, suggest KMD8 is a multi-functional molecule involved in tumor progression, In this study, we extend its oncogenic role to PCa.

## Results

### KDM8 is overexpressed in a subset of prostate tumors with high Gleason scores and its elevation drives the development of CRPC

**Fig. 1:**
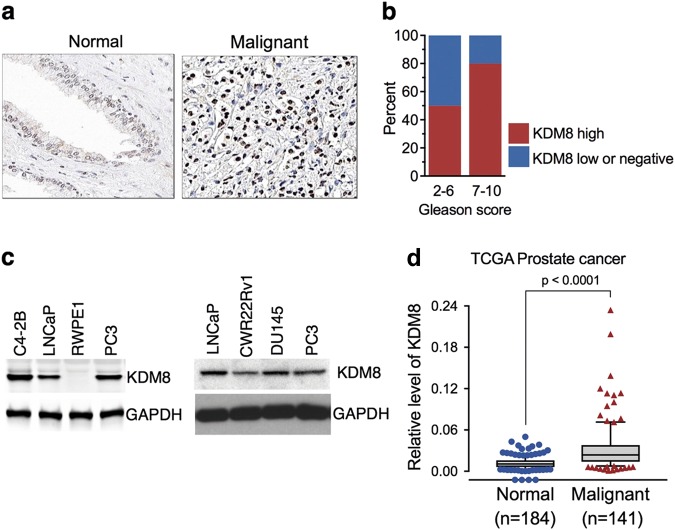
KDM8 is overexpressed in malignant prostate tissues and cancer cell lines. **a** Representative images of anti-KDM8 immunohistochemical (IHC) staining of prostate normal and malignant tissue sections. **b** Percentage of prostate cancer tumors with different Gleason scores that are scored as high or low (or negative) in the anti-KDM IHC analysis. **c** Western blot analysis of KDM8 in non-malignant RWPE1 cell line and cancerous prostate epithelial cell lines C4-2B, LNCaP, PC3, CWR22rv1, and DU145. GAPDH was used as a loading control. **d** KDM8 gene expression analysis in prostate cancer (TCGA dataset extracted from Oncomine database). Box plot derived from gene expression data in Oncomine database comparing the KDM8 expression in normal (*n* = 184) and in malignant cancer tissues (*n* = 141)

We first examined whether KDM8 expression is deregulated in tumors of PCa by immunohistochemistry (IHC) analysis. Evaluation of anti-KDM8 immunostaining of prostate specimens from 121 cases revealed that 61% of malignant tumors showed high levels of KDM8 expression while the majority of normal or non-malignant prostate tissues displayed no detectable or low levels of KDM8 (Fig. 1a, b, Table S1). Approximately 80% of tumors with high Gleason score (7–10) had positive KDM8 staining with high IHC scores, while about 25% of low (2–6) Gleason tumors displayed high KDM-IHC scores (Fig. 1b).

Similar to the expression pattern seen in prostate tissues, higher levels of KDM8 protein were detected by immunoblotting in PCa cell lines as compared to immortalized prostate epithelial cell line RWPE1 (Fig. 1c). In agreement with our own screening results, the TCGA transcriptome dataset extracted from Oncomine database (www.oncomine.com) also showed a higher expression of KDM8 in malignant PCa (*p* < 0.0001) (Fig. 1d). These data suggest KDM8 may be involved in the development of castration and therapy resistance.

**Fig. 2:**
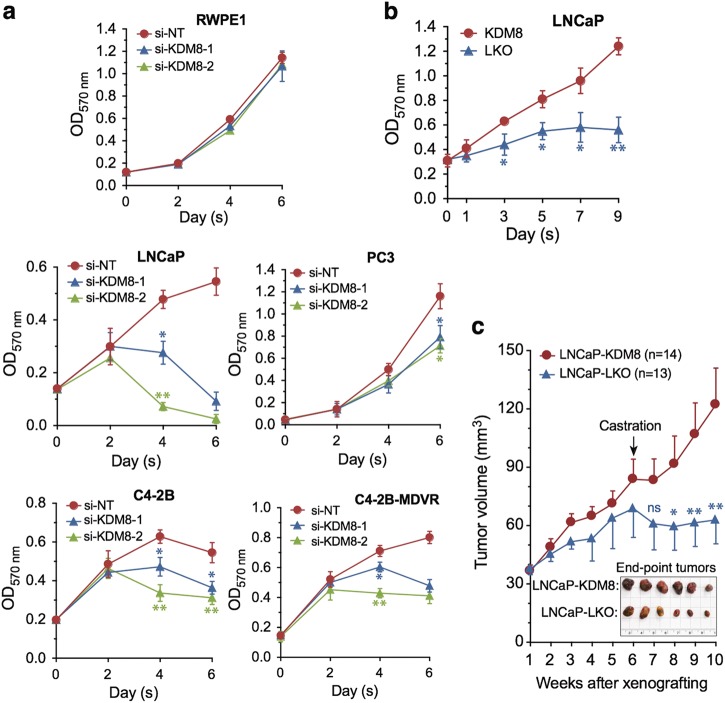
KDM8 is required for AR-positive prostate cancer cell proliferation and survival, and promotes androgen-independent cell proliferation and tumor growth. **a** Growth curves of non-malignant cell line RWPE1 and prostate cancer cell lines LNCaP, PC3, C4-2B, and C4-2B-MDVR, an enzalutamide-resistant cell line. Cells were transfected with small interfering (si)-RNAs targeting KDM8 or si-non-targeting (NT) control. Every 2 days after transfection, cell proliferation was measured by MTT assay. **b** Growth curves of LNCaP cells infected with KDM8 overexpressing (KDM8) or control (LKO) lentiviruses followed by maintaining in androgen-deprived media. Two days later infection, cell proliferation was measured by MTT assay at indicated times. **c** Tumor growth curves of KDM8 overexpressing and LKO control LNCaP cells in xenografting mouse model. Cells were injected subcutaneously into the dorsal flanks of athymic nude mice (8 mice per group) and tumor volumes were measured every week by using calipers. Tumor-bearing mice were also castrated at indicated time point. Insert: photograph of xenograft tumors. The quantitative data shown in **a** and **b** are the mean ± S.D. of three separate experiments. The average tumor volumes are presented as the mean ± S.E.M. *p < 0.05; **p < 0.01, by paired Students’ *t* (MTT assay) or ANOVA test (xenografting study)

Consistent with its role in the control of PCa cell proliferation and survival, knockdown of KDM8 inhibited the proliferation of both LNCaP, C4-2B and C4-2B-MDVR, an enzalutamide-resistant variant of C4-2B, and, to less extent, the AR negative PC3 cells. Importantly, KDM8 knockdown had little effect on the non-malignant prostate cell line RWPE1 (Fig. 2a and Figure S2). By contrast, overexpression of KDM8 in RWPE1 cells stimulates the growth of this cell line (Figure S1). These data suggest that KDM8 is related to malignant cell growth. We then asked whether overexpression of KDM8 can convert an androgen-sensitive LNCaP into androgen-independent. Accordingly, KDM8 was overexpressed in LNCaP cells via lentivirus infection and cultured in androgen-deprived conditions. As shown in Fig. 2b and Figure S3a, KDM8 overexpression markedly stimulated androgen-independent cell proliferation of LNCaP cells.

These studies were then extended to in vivo tumorigenesis assay. KDM8 overexpressing and vector control LNCaP cells (Figure S3b) were injected into athymic nu/nu mice and the tumor growth was monitored. In intact animals, the KDM8-overexpressing LNCaP grew slightly faster than vector- infected LNCaP (LNCaP-LKO). Upon castration, LNCaP-KDM8 tumors continued to grow whereas LNCaP-LKO ceased to do so (Fig. 2c). Together, these data suggest that elevated KDM8 expression is related to malignant transformation of PCa cells and has the potential to cause castration-resistance.

### KDM8 regulates tumor metabolism via partnership with PKM2

#### KDM8 translocates PKM2 into nucleus

**Fig. 3:**
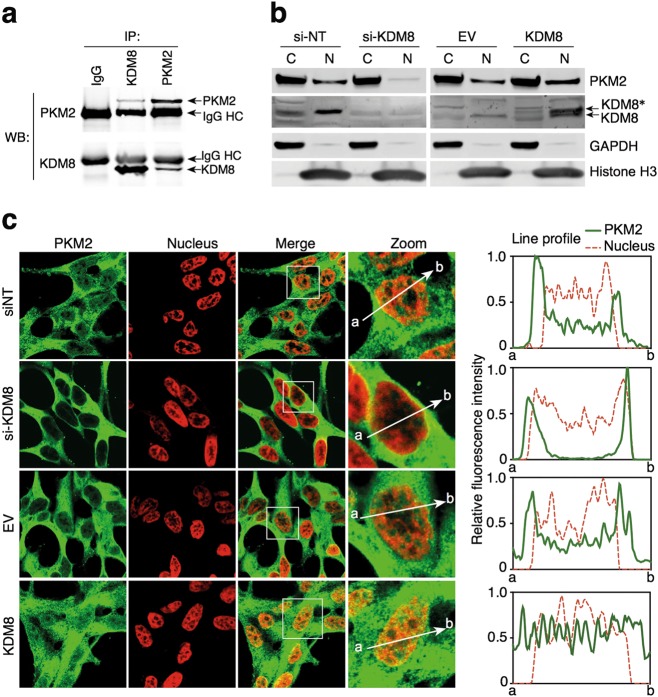
KDM8 regulates PKM2 nuclear translocation. **a** Interaction of endogenous KDM8 and PKM2 in LNCaP cells. Reciprocal immunoprecipitation (IP) and immunoblotting (IB) were performed with PKM2 and KDM8 antibodies as indicated. **b** Subcellular localization of KDM8 and PKM2 in LNCaP cells. Nuclear (Nuc) and cytosolic (Cyto) fractions were prepared from LNCaP cells transfected with si-NT, si-KDM8, EV, or KDM8-expressing vector, followed by immunoblotting analysis with antibodies as indicated. KDM8*, Flag-tagged KDM8. **c** Confocal immunomicroscopy analysis of PKM2 nuclear translocation. Treated cells were fixed and immunostained with anti-PKM2 (PKM2) and 4′,6-diamidino-2-phenylindole (DAPI, nucleus), respectively. The framed regions marked in the merged images (Merge) are zoomed at the next (Zoom). The line profiles of PKM2 and DAPI signals were measured by ZEN 2011 (Carl Zeiss, Germany) software. Scale bars, 10 µm

One of the hallmarks of aggressive PCas including castration and therapy resistant is the metabolic adaptation, where aerobic glycolysis dominant over mitochondria oxidative phosphorylation [1, 2]. Previously, we reported that in breast cancer, a novel function of KDM8 is its association with PKM2 and its ability to translocate PKM2 into nucleus to become a coactivator of HIF-1α to transcriptionally activate glycolytic genes in favor of Warburg effects [7]. We therefore asked whether KDM8 is able to modulate the tumor metabolism in PCa cells. First, in a reciprocal immunoprecipitation analysis, we showed that KDM8 and PKM2 associate with each other in LNCaP cells (Fig. 3a). Furthermore, in both cell fractionation and confocal microscopy analyses, KDM8 overexpression enhances the translocation of PKM2 into the nucleus (Fig. 3b, c). Conversely, knockdown of KDM8 reduces PKM2 translocation (Fig. 3b, c). The nuclear translocation studies were aided by confocal microscopy (Fig. 3c) where the fluorescent intensity of PKM2 across the nucleus was traced as illustrated on the right panel and the average intensity of counting 10 nuclei for KDM8 overexpressing cells measured.

#### KDM8/PKM2 upregulates glycolytic genes

**Fig. 4:**
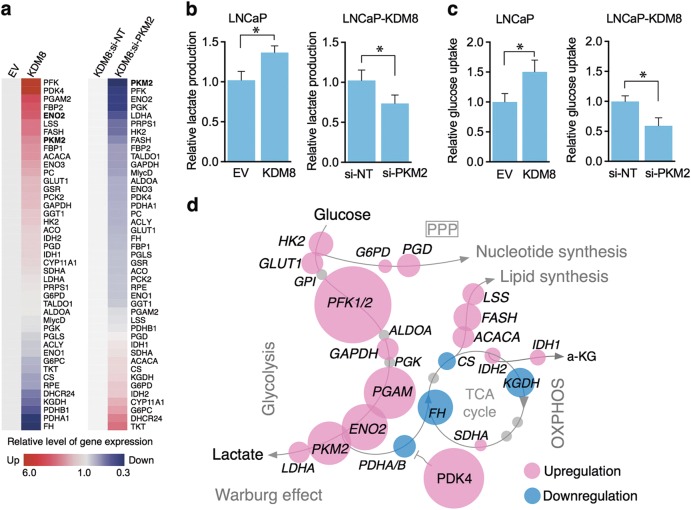
Overexpression of KDM8 reprograms glucose metabolism in prostate cancer cells. **a** Heat map of metabolic gene expression in KDM8-overexpressed LNCaP cells (left panel) and the KDM8-overexpressed LNCaP cells knocking down with siRNA targeting PKM2 (right panel). The gene expression levels were measured by qRT-PCR. Data were analyzed as fold change as compared to the EV control (left panel) or si-NT after normalizing to internal control, 16S rRNA. **b** Measurements of the levels of extracellular lactate and **c** glucose update in EV control and KDM8-overexpressed LNCaP cells. Data are expressed as mean ± S.D. with three separate experiments. *p < 0.05. **d** A schematic diagram of glucose metabolic flux. Overexpression of KDM8 induces redirection of metabolic flux into the biomass synthesis pathways. The affected genes are marked in red (upregulated) or in blue (downregulated). The sizes of the circles are proportional to the expression levels of the genes indicated

A consequence of the KDM8-mediated PKM2 nuclear translocation is the upregulation of glycolytic and metabolic genes, the targets of HIF-1α [7]. The genes involved in glycolysis upon KDM8 overexpression shown in Fig. 4a (GLUT1, HK2, PKM2, LDHA, etc.) are all upregulated, whereas genes involved in mitochondrial pyruvate flow such as PDHA1 and PDHB1 are down-modulated. These data are summarized in the bubble diagram of Fig. 4d. The overall consequence is the switch of mitochondria oxidative phosphorylation to glycolysis [7]. Indeed, in LNCaP-KDM8 cells, the uptake of glucose (Fig. 4b) and lactate production (Fig. 4c) are increased over the control cell lines, and they are PKM2-dependent, as siRNA targeting PKM2 reverse the effects. To demonstrate that the heightened expression of glycolytic genes by KDM8 overexpression is mediated by PKM2, we knocked down PKM2 in LNCaP-KDM8, and monitored their expression levels (Fig. 4a, right panel and Figure S4). Many of the genes overexpressed in LNCaP-KDM8 are indeed down-modulated in the absence PKM2. Taken together, these data suggest that KDM8 is a major metabolic regulator, in partnership with PKM2, forms a complex that translocates into the nucleus to reprogram gene expression toward aerobic glycolysis (Warburg effect) (Fig. 4d).

### KDM8 regulates androgen responses via partnership with AR

#### KDM8 activates AR response in the absence of androgen

**Fig. 5:**
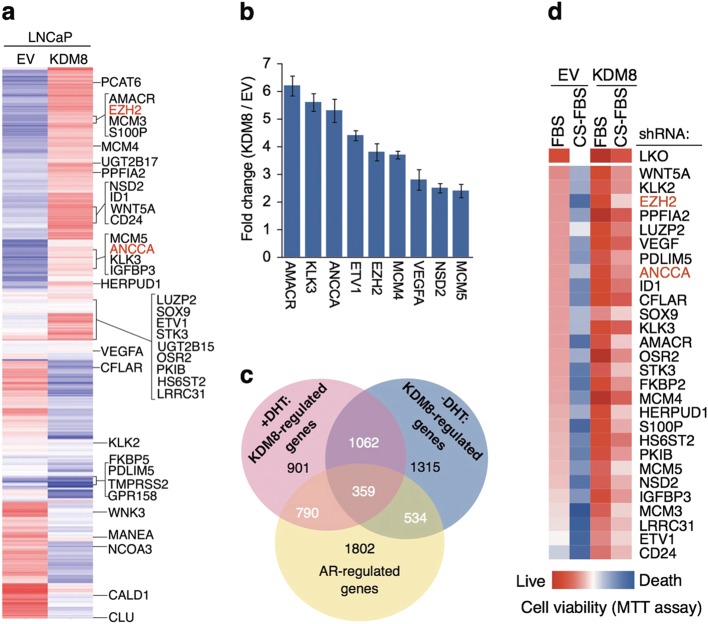
KDM8 controls specific subsets of androgen-responsive program. **a** Heat map of hierarchical clustering of androgen-regulated genes (ARGs). LNCaP-overexpressed KDM8 and EV control cells were cultured in the presence or absence of 1 nM DHT followed by gene expression profiling with microarray analysis. The ARGs that differentially expressed by KDM8 (≥1.5-fold) and the positions for well-known ARGs are indicated. Two genes EZH2 and ANCCA selected for ChIP assay are marked in red. **b** qRT-PCR analysis of the selected ARDs. LNCaP cells (KDM8 and EV) were cultured in the androgen-deprived media for 3 days followed by qRT-PCR analysis. Triplicate experimental data were expressed as fold change as compared to EV control after normalizing to internal control 16S rRNA. **c** Venn diagram showing the overlap of KDM8-regulated genes and ARDs. **d** Heat map showing cell viability of LNCaP cells (KDM8 and EV) by knocking down the indicated ARGs in the absence of androgen. LNCaP cells infected with the shRNAs targeting the ARGs as indicated were then cultured in media containing normal FBS or charcoal-stripped FBS (CS-FBS) for 3 days. Cell viability was assessed by MTT assay. EZH2 and ANCCA selected for chromatin immunoprecipitation (ChIP) assay are marked in red

In addition to metabolic adaptation, aberrant activation of AR responses is another critical step of castration-resistance. In our transcriptome analysis of KDM8 overexpressing cells (microarray data were deposited in NCBI GEO database accession number: GSE56908), unsupervised clustering analysis (Fig. 5a) indicated that among the genes modulated by KDM8 (in the absence of DHT), a significant fraction corresponds to androgen response genes, which include AMACR, EZH2, ANCCA, KLK3, and NSD2/WHSC1, suggesting KDM8 overexpression leads to aberrant activation of AR. Real-time RT-PCR validated the microarray results (Fig. 5b). As shown in the Venn diagram (Fig. 5c), a significant fraction of genes (27% in the absence of DHT and 37% in the presence of DHT) with altered expression (2×) elicited by elevated KDM8 overlap with androgen-responsive genes (ARG).

#### Genes involved in androgen-independent growth

To demonstrate that the KDM8-upregulated genes are functionally relevant in the induction of androgen-independence, we individually knocked down the upregulated genes (Figure S6) and monitor the growth of LNCaP-KDM8 in regular and in charcoal-dextran media (Fig. 5d). In regular media, the individual knockdowns had moderate effects (dark red to light red), while in charcoal-stripped media, as expected, the LNCaP-EV cells were not growing well, with individual knockdown of many of the genes including EZH2 caused severe effects. In LNCaP-KDM8, the effects of individual gene knockdown of on growth are generally minimal. In charcoal-stripped media, the inhibitory effects on growth of individual knockdown of genes such as EZH2 were again detected. These results suggest that KDM8 induced genes (in the absence of androgen) were indeed relevant to androgen-independent growth, and they jointly contribute to the growth and survival of LNCaP under androgen-deprived conditions.

### KDM8 associates with AR and acts as a novel AR coactivator

#### KDM8 associates with and transactivates AR

**Fig. 6:**
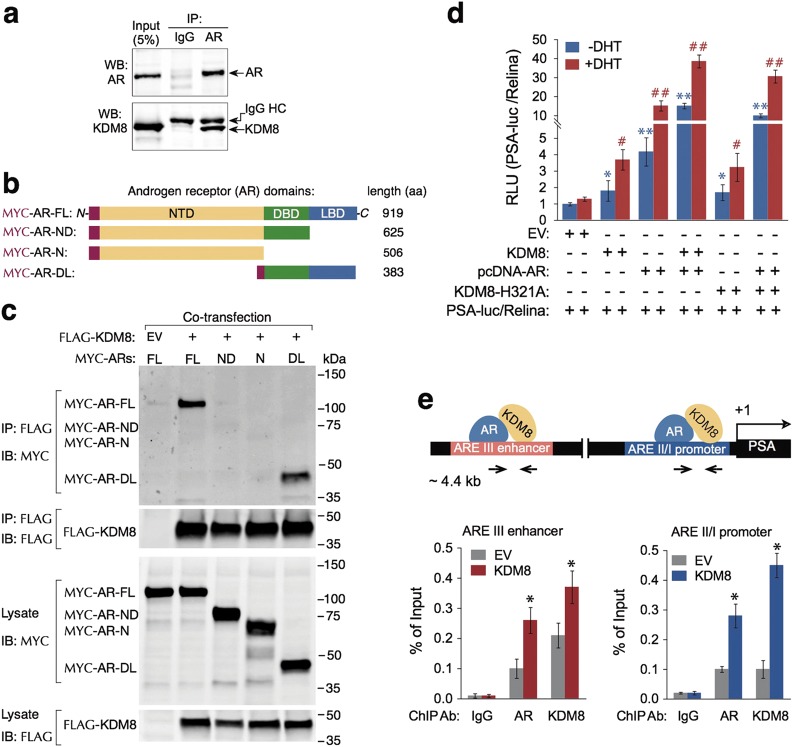
KDM8 acts as a novel coactivator of AR. **a** Interaction of endogenous KDM8 and AR in LNCaP cells. Reciprocal immunoprecipitation (IP) and immunoblotting (IB) were performed with PKM2 and AR antibodies as indicated. Input indicates non-immunoprecipitated cell lysates. **b** Schematic diagram of different MYC-AR expression vectors. NTD N-terminal domain, DBD DNA-binding domain, LBD ligand-binding domain, MYC-AR-FL (full length), MYC-AR-ND, NTD + DBD; MYC-AR-N, NTD; MYC-AR-DL, DBD + LBD. **c** Co-IP and IB of FLAG-KDM8 with different Myc-AR constructions with anti-M2-Flag and Myc antibodies as indicated. Non-immunoprecipitated samples are indicated as lysate. **d** PSA promoter activity in prostate cancer cell line PC3. Dual luciferase assays were performed in PC-3 cells transfected with PSA-luc/TK-rellina (20:1) combining with the expression vectors of AR and KDM8 (wild-type and mutant H321A) as indicated. At 24 h after transfection, the cells were cultured in RPMI-1640 medium with CS-FBS for 6 h prior to treatment with 10 nM DHT for 8 h before measurement of the luciferase activity. The relative luciferase activity (RLU, relative light unit) was calculated by their normalized luciferase activity to the EV control with (red bar) or without DHT (blue bar) stimulation. Error bars represent as mean ± S.D. from three different experiments. ^#^^,^ *p < 0.05, ^##^^,^ **p < 0.01, by paired Students’ *t*-test. **e** ChIP assay of KDM8 and AR binding to the PSA enhancer/promoter regions in LNCaP cells. (Upper) Schematic diagram of the positions of PSA enhancer and promoter. The distances of the enhancer and promoter from transcription starting site (+1) as well as AR and KDM8 are presented. Primer sets used for qPCR are indicated. (Lower) ChIP-qPCR analysis of the occupancy of AR and KDM8 at PSA promoter and enhancer regions. LNCaP cells maintained in androgen-deprived media were used for the preparation of immunoprecipitated genomic DNA with AR or KDM8 antibody as indicated. Data were expressed relative to relevant IgG control. Data are shown as mean ± S.D. of three independent experiments. *p < 0.05, by paired Students’ *t*-test

Given that a majority of ARGs were regulated by KDM8, we asked whether KDM8 physically associates with AR and serves as a coactivator [33, 34]. We first performed co-immunoprecipitation assay. As shown in Fig. 6a, anti-AR antibody, but not the control IgG, effectively co-precipitated KDM8, demonstrating that indeed KDM8 specifically formed complexes with AR. To further characterize the interaction, we expressed Flag-tagged KDM8, together with Myc-tagged AR full-length (FL) or its deletion mutants: N (N-terminal domain, NTD), ND (NTD plus DNA-binding domain, DBD), or DL (DBD plus ligand-binding domain LBD) in 293T cells and performed immunoprecipitation with Flag-tagged KDM8, and detected strong association of KDM8 with DBD-LBD but not with the NTD (Fig. 6b). The mapping data suggest that the interaction domain of AR resides in the ligand-binding domain.

To determine whether KDM8’s interaction with AR affects the transcriptional regulation activity of AR, we performed reporter gene assays with PSA gene enhancer and promoter-linked luciferase. Figure 6c showed that although KDM8 alone slightly induced the reporter activity, co-expression of KDM8 and AR markedly increased the reporter activity from 4-fold (AR alone) to 15-fold (AR + KDM8) and from 15-fold (AR alone) to 38-fold (AR + KDM8) in the absence and presence of DHT, respectively. Interestingly, changes in the amino acid sequence in the Jumonji domain (H321A), which inactivates its demethylase activity, only partially reduced the coactivation of AR, indicating the KDM8 behaves in both demethylase dependent and independent manner.

#### KDM8 is corecruited with AR to PSA promoter and enhancer

To study whether KDM8 functions as an AR coactivator in vivo, we studied the co-recruitment of KDM8 and AR at the enhancer and promoter regions of PSA by ChIP assays (Fig. 6d). In parental LNCaP, KDM8 was seen to occupy both the enhancer and promoter region of PSA locus under androgen-deprived conditions. In KDM8 overexpressing LNCaP, increased occupancy of KDM8 was detected. The level of AR also increased under these conditions, suggesting that KDM8 facilitates the recruitment of AR to the target sites. Together, these results demonstrate that KDM8 can act as a novel AR coactivator through its direct interaction with AR.

### KDM8 mediates AR activation of EZH2 via ANCCA to stimulate PCa cell growth

**Fig. 7:**
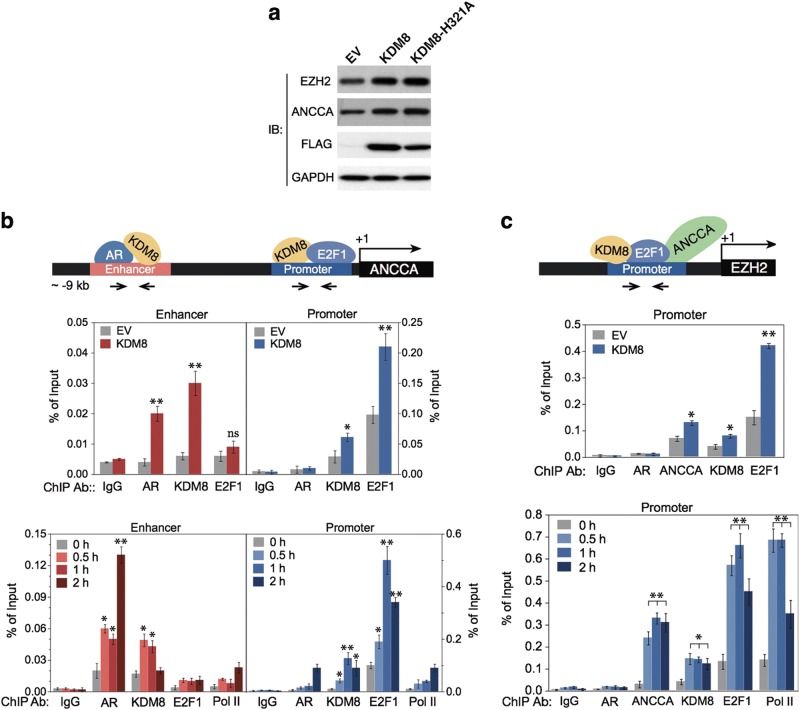
KDM8 facilitates AR and E2F1 recruitment to the ANCCA and EZH2 promoters and upregulation of ANCCA and EZH2 gene expressions. **a** Immunoblotting assay of EZH2 and ANCCA in the KDM8-overexpressing LNCaP cells. Cells transfected with FLAG-tagged wild-type KDM8 or mutant KDM8-H321A were analyzed by immunoblotting (IB) with the indicated antibodies. GAPDH was used as a loading control. **b** ChIP analysis of the association of KDM8, AR, and E2F1 binding to ANCCA enhancer/promoter in LNCaP cells. (Upper) Schematic diagram of the positions of ANCCA enhancer and promoter. The transcription starting site is marked as +1. (Lower) ChIP-qPCR analysis of the occupancy of AR/KDM8 and KDM8/E2F1 at ANCCA enhancer and promoter, respectively. The immunoprecipitated chromatins were prepared from LNCaP cells maintained in androgen-deprived media followed by immunoprecipitation with indicated antibodies. Data were expressed as percent immunoprecipitation relative to input chromatin. Data are shown as mean ± S.D. of triplicate experiments. *p < 0.05, Student’s *t*-test. **c** ChIP analysis of the association of KDM8, E2F1, and ANCCA binding to EZH2 promoter in LNCaP cells. (Upper) Schematic diagram of the position of EZH2 promoter. The transcription starting site is marked as +1. (Lower) ChIP-qPCR analysis of the occupancy of KDM8/E2F1/ANCCA at EZH2. The immunoprecipitated chromatin was prepared from LNCaP cells maintained in androgen-deprived media followed by immunoprecipitation with the indicated antibodies. Pol II, RNA polymerase II. Data were expressed as percent immunoprecipitation relative to input chromatin. Data are shown as mean ± S.D. of triplicate experiments. *p < 0.05, Student’s *t*-test

To further validate KDM8 as a critical coactivator of AR in prostate carcinogenesis, we wish to identify the recruitment of KDM8 to the promoters of AR target genes involved in tumor progression. We have selected EZH2 pathway to illustrate the role of KDM8. EZH2 is among the genes whose knockdown have the most severe effects on androgen-independent growth and that there is strong evidence that EZH2 is involved in PCa progression (see Discussion). We previously showed that EZH2 is activated by AR via ANCCA, a chromatin remodeling ATPase and a coactivator of E2F1 [35], which is also upregulated by KDM8 overexpression (Fig. 7a) (Supplementary Information Figure S1b). Here, we show that KDM8 is directly involved in both ANCCA and EZH2 activation in LNCaP cells. We first demonstrated that KDM8 was corecruited with AR to the enhancer region of ANCCA locus in both the control (EV) and KDM8 overexpressor (KDM8) (Fig. 7b, upper panel). The amount of AR recruitment increases with KDM8 overexpression, suggesting that KDM8 as a coactivator facilitates AR targeting to chromatin. Furthermore, the co-recruitment of KDM8 and AR were induced by androgen in a time-dependent manner (Fig. 7b, lower panel), followed by the recruitment of RNA polymerase II (Pol II) for transcription. The elevated expression of ANCCA lead to an increased accumulation of ANCCA and its transcriptional partner E2F1 near the promoter of EZH2 [35], as seen in KDM8 overexpressing cells (Fig. 7c, upper panel). Importantly, we found KDM8 is also recruited to this site in a time-dependent manner (Fig. 7c, lower panel). Finally, to validate the roles of ANCAA and EZH2 in KDM8-mediated growth, these genes were knocked down in LNCaP-KDM8 (Supplementary Information Figure S7a, c), and cell growth was significantly diminished in a manner similar to KDM8 knockdown (Supplementary Information Figure S7b, d). Taken together, these results confirmed KDM8’s role as a coactivator of AR and is involved in the activation of EZH2.

### Targeting KDM8 in therapy-resistant PCa cells

**Fig. 8:**
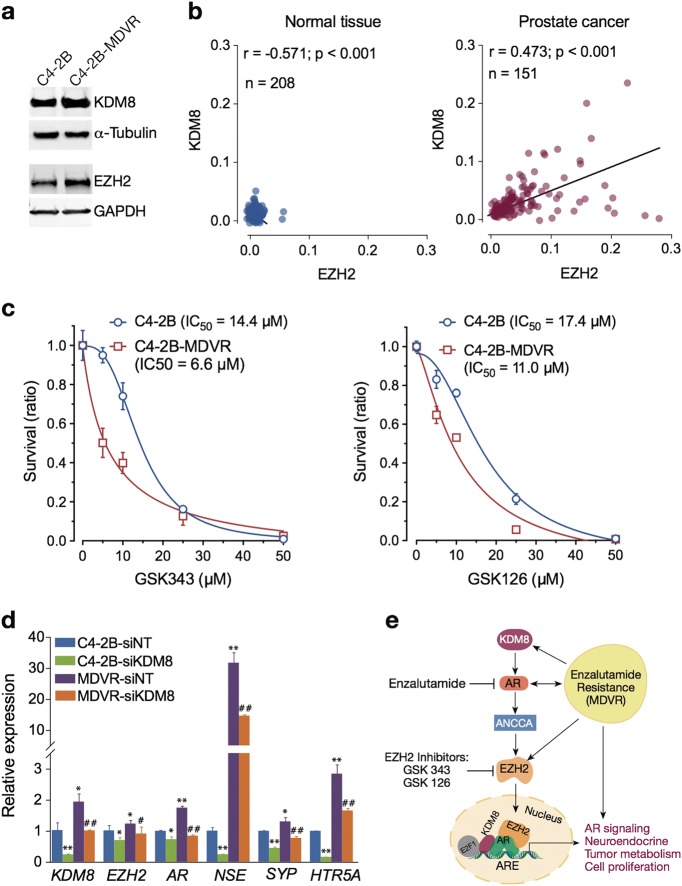
Overexpression of KDM8 confers upregulation of neuroendocrine markers and renders prostate cancer cells more sensitive to killing by EZH2 inhibitors. **a** Immunobloting assay of KDM8 expression in C4-2B cell line and its enzalutamide-resistant derivative, C4-2B-MDVR. α-tubulin and GAPDH were used as a loading control. **b** Correlation analysis between gene expression levels of KDM8 and EZH2 in prostate cancer patients (right panel) and normal controls (left panel) from TCGA dataset extracted from Oncomine database. Pearson’s correlation (*r*) values are shown in each graph. **c** Cell survival curves of C4-2B and C4-2B-MDVR cells treated with EZH2 inhibitors. Cells were exposed to the EZH2 inhibitors GSK343 and GSK126 in different doses from 0 to 50 μg/ml for 72 h followed by MTT assay. Data were expressed as the mean ± S.D. of triplicate experiments. The values of IC_50_ were calculated and shown. The regression lines represent the fit to a non-linear regression model using GraphPad Prism. **d** qRT-PCR analysis of KDM8, EZH2, AR, and neuroendocrine markers mRNA expression. Parental cell lines (C4-2B) and the enzalutamide-resistant derivative (C4-2B-MDVR) were transfected with si-RNAs targeting KDM8 (si-KDM8) or non-targeting control (si-NT) for 48 h. The relative mRNA levels of the above genes were normalized to 18S rRNA. Data were represented as the mean ± S.D. of triplicate experiments. Values in C4-2B cells transfected with si-NT were set to 1. *^,#^*p* < 0.05, **^, ##^*p* < 0.01, by paired Students’ *t*-test. **e** Model of KDM8-driven CRPC and neuroendocrine markers expression via AR–EZH2 axis

There have been significant interests in developing therapies overcoming castration as well as enzalutamide resistance. We have developed an enzalutamide-resistant C4-2B cell line, C4-2B MDVR [36]. As shown in Fig. 8a, C4-2B MDVR has a heightened expression of KDM8, accompanied by elevated expression of EZH2, as compared to the enzalutamide-sensitive C4-2B. TCGA public data analysis revealed a general positive correlation of KDM8 and EZH2 in PCa cells (Fig. 8b). Both the in vitro (Fig. 2a) and in vivo (Figure S8) growth of C4-2B-MDVR is inhibited by KDM8 knockdown. We show here that C4-2B-MDVR is sensitive to cell killing by two EZH2 inhibitors (GSK343 and GSK126) (Fig. 8c). Interestingly, C4-2B-MDVR is more sensitive to EZH2 inhibitor than its C4-2B, despite the higher EZH2 expression level. This suggests that during the selection of enzalutamide resistance, C4-2B-MDVR has developed a reliance on the EZH2 pathway. One potential mechanism is the adaptation of neuroendocrine phenotypes, caused by EZH2 overexpression [37]. Indeed, in C4-2B-MDVR cell line, the expressions of neuroendocrine marker genes (NSE, SYP, and HTRSA) are elevated over C4-2B (Fig. 8d). Moreover, siRNA knockdown of KDM8 significantly decreased the expression of neuroendocrine genes, suggesting that the KDM8–AR–EZH2 axis may be involved in the generation of neuroendocrine phenotypes (Fig. 8e), thereby conferring resistance to enzalutamide. EZH2 inhibitor or KDM8 knockdown reverses this trend and resensitizes these cells toward enzalutamide.

## Discussion

Androgen ablation therapies, especially with the next-generation anti-androgen ezalutamide and androgen synthesis blocker abiraterone, are effective when cells are dependent on androgen for their growth. Additional therapies and molecular targets are being sought to help targeting PCa cells which either harbor aberrantly activated, androgen-independent AR or constitutively activated oncogenes bypassing the need of AR signaling. In this study, we show that KDM8 could be such a target as it is involved in aberrant AR as well as AR-independent signaling by being a partner for both androgen receptor and PKM2. Androgen receptor and PKM2 are both engines for PCa growth, as the former regulates cell cycle, and the latter, tumor metabolism. Adaptation to tumor metabolism is also important for tumor cells to escape the nutrition deprived conditions during therapeutic intervention. Indeed, Gene set enrichment analysis (GSEA) in KDM8-high prostate clinical samples several metabolic pathways including those involved in lipid, glycolysis, and pyruvate are enriched (Figure S9). Thus, targeting KDM8 should not only thwart castration-resistance but may also reduce therapy resistance.

### KDM8 as a coactivator of PKM2

PKM2 is a pivotal enzyme in determining the metabolic flow to lactate, TCA cycle, or biosynthetic pathway [38]. For tumor cells, PKM2 enzymatic activity is required, but should be maintained at a balanced level, high enough to produce sufficient pyruvate, but low enough to accumulate enough glycolytic precursors for the biosynthesis of macromolecules. PKM’s activity can be modulated by a variety of ways including metabolite binding [39, 40] and post-translational modifications [41–44]. An additional way is to divert some of the cytosolic PKM2 into nucleus where it serves as a coactivator to transcriptionally activate more metabolic genes [6, 45, 46]. The mechanisms associated with PKM2 translocation are multiple, including phosphorylation, [47] acetylation [43], and hydroxylation [45]. Here, we describe another way whereby KDM8 binds PKM2 and transports PKM2 into the nucleus and enhances metabolic gene expressions. Nearly all the glycolytic enzymes have higher level of expressions to accelerate the glycolysis reactions. Many of these genes are targets of HIF-1a. Their upregulations depend on the presence of both KDM8 and PKM2, as knockdown of either one diminished the effects.

### KDM8 as a coactivator of AR

KDM8 is overexpressed in nearly all PCa cell lines and in a significant portion of high-grade PCas. Its overexpression leads to AR activation under androgen-deprived conditions and confers castration-resistance in xenograft model. Knockdown of KDM8 preferentially affected the growth of PCa cells with virtually no effects on normal prostate epithelial cell. We showed that KDM8 is a bona fide AR coactivator by demonstrating its association with AR, synergistic activation of ARE-driven promoters with AR, and co-recruitment with AR to the target sites. The extensive overlap of KDM8 and AR-activated genes lends credence to the notion that KDM8 is a natural coactivator of AR. At present, we do not know how KDM8 enhances AR activity. Our data suggest that it facilitate the delivery of AR to the chromatin targets, likely due to KDM8’s ability to modulate the chromatin conformation. In this regard, we found that the demethylase activity for H3K36me3, however, is not absolutely required for co-activating AR. It is possible the newly discovered cleavage activity may modulate the chromatin structure without demethylase activity [27, 28].

### KDM8/AR signaling network

Overexpression of KDM8 leads to castration-resistance as determined by in vitro culture and in vivo xenograft model and the upregulation of a subset of AR responsive genes. Many of these genes have been reported to be important factors in castration-resistance. We wished to determine which of them are important downstream effectors of KDM8-mediated castration-resistance. Based on an unbiased siRNA screening, we found that all individually contributed to some extent to the growth and survival phenotypes under androgen-deprived conditions, with the knockdown of EZH2, AMACR, NSD2, MCM3, ETV1, and CD24 giving the most severe consequence. We chose EZH2 to further define the detailed mechanisms of upregulation, because of its well-recognized role in castration-resistance [48, 49], EMT/metastasis [50, 51], chemoresistance [52], and neuroendocrine phenotypes [53] of PCa. EZH2, the catalytic subunit of the polycomb repressive complex PRC2, is a general repressor of gene transcription and shown to be a corepressor of androgen receptor in PCa [54]. Interestingly, in castration-resistant PCa, EZH2 also acts as a coactivator to modulate genes involved in castration-resistance [55]. EZH2 is commonly overexpressed in PCas [48] with the highest expression in lethal-type castration-resistance PCa [56]. Our studies provide additional insights into the regulation of the EZH2 in PCa. Our data suggest that KDM8 and ANNCA are recruited to the promoter of EZH2 and regulate its expression. Our data confirmed previous results showing ANCCA is a direct target of AR and further indicate that KDM8 also participates in the transcription of this gene.

### KDM8/EZH2 and enzalutamide resistance

The second-generation anti-androgen enzalutamide has improved the prospects of castration-resistance patients. However, enzalutamide-resistant tumors eventually emerge, leading to mortality. Some of the resistant cells, especially those with RB, p53, and PTEN defects, is marked by an intermediate phenotype with the expression of both androgen receptor and neuroendocrine markers [37]. EZH2 or polycomb complex was shown to be an inducer of the neuroendocrine phenotypes [53, 57]. Inhibition of EZH2 reverses the phenotypes and resensitize the resistant cells toward enzalutamide [37]. As KDM8 is an upstream regulator of EZH2 as shown in this study, we checked whether heightened KDM8 expression is associated with enzalutamide resistance. In the enzalutamide-resistant C4-2B-MDVR line we developed [1], KDM8 expression was elevated, which is accompanied by upregulation of neuroendocrine genes. These clones are more sensitive to EZH2 inhibitor and KDM8 knockdown reduces neuroendocrine markers, overcoming enzalutamide resistance. These data suggest that KDM8 may have a role in therapy resistance as well.

In summary, we have identified KDM8 as a dual coactivator of AR and PKM2, which drives PCa growth and provides needed metabolic energy. These properties, together with its ability to activate EZH2, a factor known to be involved in castration and therapy resistance, make KDM8 a promising therapeutic target for PCa.

## Materials and methods

### Cell lines and cell culture

RWPE1, LNCaP, PC3, C4-2B, DU145, CWR22V1, and HEK293 cells were obtained from the American Type Culture Collection (ATCC, Manassas, VA). C4-2B cells resistant to enzalutamide (C4-2B-MDVR) were generated by culturing C4-2B cells to increasing concentrations of enzalutamide (5–40 µM) in media for over 12 months and maintained in complete media supplemented with 20 µM enzalutamide as described previously [58, 59]. HEK293 cells were cultured in complete DMEM medium. RWPE1, LNCaP, LNCaP-KDM8 [17], PC3, C4-2B, DU145, and CWR22RV1 cells were cultured in RPMI-1640 medium supplied with 10% heat-inactivated FBS, 100 U/ml penicillin, and 0.1 mg/ml streptomycin in a humidified incubator with 5% CO_2_. For hormone induction experiments, the growth media were replaced by RPMI-1640 containing 10% charcoal-dextran-treated (CDT) FBS (CDT-FBS) for 2 days before treatment with 10 nM dihydrotestosterone (DHT).

### Cell proliferation and survival assays

Cells were seeded in 48-well plates in RPMI-1640 complete medium one day before KDM8 knocking down experiments. After incubation for 24 h, the cells were then transfected with si-RNAs of non-targeting control (si-NT) or specific targeting KDM8 (si-KDM8#1 and si-KDM8#2). The cell proliferation was measured every 2 days by MTT colorimetric assay according to the manufacturer’s instruction (Roche, IN). C4-2B and C4-2B-MDVR cells were seeded on 96-well plates at a density of 1 × 10^4^ cells per well in RPMI-1640 media containing 10% FBS and treated with different concentrations of EZH2 inhibitors (GSK343 and GSK126) for 48 h. Cell viability was determined by the MTT colorimetric assay and the cell survival rate (%) was calculated as cell survival rate (%) = (OD570 nm of treatment group/OD570 nm of control group) × 100%.

### PSA-luciferase activity assay

The PSA-luciferase activity assay in PC-3 cells was as described previously [60].

### Co-immunoprecipitation and immunoblotting analysis

Co-immunoprecipitation was performed using cell lysates from LNCaP and 293T cells for endogenous and ectopically expressed proteins, respectively, for investigating protein–protein interaction has been described [7]. The immunoblotting assay was performed using the following antibodies: Flag-M2, Myc-tag (Cell Signaling), KDM8 [17]; β-actin (Sigma-Aldrich); PSA (Santa Cruz Biotech); ANCCA [61], EZH2 (Cell signaling), and GAPDH (Santa Cruz Biotech).

### Chromatin immunoprecipitation assay

ChIP assay was performed as described previously [61, 62]. The precipitated chromatin DNA was analyzed by real-time PCR with SYBR green on an iCycler instrument (BioRad) with gene specific primer sets (Supplementary Information).

### Microarray assay

LNCaP cells overexpressing KDM8, KDM8-H321A, or EV were grown in 10 cm dishes, and total RNAs were extracted from 80% confluent cell using TRIzol (Invitrogen) extraction according to the manufacturer’s instructions. Microarray gene expression profiling was performed by 3′ IVT expression analysis with Affymetrix GeneChip Human Genome U133 Plus 2.0 Arrays. The KDM8-responsive and androgen-responsive gene expression changes were identified by pairwise comparison analyses (≥1.5-fold threshold), and expression patterns were analyzed by hierarchical clustering.

### Mouse xenograft tumor study

Xenograft tumor studies were conducted utilizing the 6-week-old male athymic Nu/Nu mice (Harlan). The total number of mice (16) was randomly divided into two sets of 8 each. Mice of the two sets were inoculated subcutaneously with one million of LNCaP cells overexpressing KDM8 or vector control (EV) in 100 μl of 50% Matrigel (BD Biosciences), respectively. Tumor growth was monitored and the length (L), width (W), and height (H) measurements taken every 7 days. The tumor volume was calculated using the formula (L × W × H) × 0.52. For castration study, the tumor-bearing mice were castrated when the tumors reached their, respectively, peak volumes (6-week post injection). All mice were killed by 10 weeks post injection. The animal tumor studies were approved by National Health Research Institutes Institutional Animal Care and Use Committee (approval number: NHRI-IACUC-102087) and carried out under the institutional guidelines with animal welfare standards.

### IHC and statistics analysis

IHC was performed as described previously [62] with the following modifications. Two tissue microarrays from US Biomax were used, which were TMA PR751 containing 73 IHC-scorable cores from 73 cases and TMA PR952 containing cores from 48 cases, with normal prostate tissues adjacent to tumors and Gleason scores. The slides were then incubated with anti-KDM8 rabbit polyclonal antibody (homemade) at 1:100 dilutions overnight at 4 °C, followed by incubations with biotinylated secondary antibody and the ABC reagents in the Vectastain Elite kit and counter-stained with hematoxylin. The percentage of positive nuclear staining was scored as follows: 0–<5%, scored as KDM8-negative or non-detected; 5–<25%, scored as KDM-low; and >25%, scored as KDM-high. Differences and correlations in immunostaining among groups were analyzed with *χ*^2^ or Fisher’s exact test.

### Confocal microscopy analysis

Confocal microscopy analysis for assessment of PKM2 nuclear translocation was performed as described previously [7].

### Glucose uptake assay and lactate production assay

Glucose uptake assay and lactate production assay were described previously [7].

### Nuclear and cytosolic fractionation

Fractionation of nuclear and cytosolic extracts was performed by using NE-PER® Nuclear and Cytoplasmic Extraction kit (Thermo Scientific) according to the manufacturer’s instruction. Overall, 15 µl of each fraction was analyzed immunoblotting assay.

### Oncomine data analysis

KDM8 and EZH2 expression in PCa patient cohorts was extracted from Oncomine database (www.oncomine.com) [63]. In the database, the Cancer Genome Atlas (TCGA) dataset was extracted and used to compare the differences of clinical specimens between cancer and normal by using a threshold of *p* < 0.05. Statistical analysis was performed with one-way ANOVA or two-tailed *t*-tests. Correlation between EZH2 and KDM8 was assessed by using the Pearson correlation coefficient.

### Statistical analysis

Comparisons were performed with a Student’s *t*-test with *p-*values denoted as *p < 0.05 and **p < 0.01 (N.S., not significant). Graphpad Prism software (La Jolla, CA, USA) was used to calculate mean and standard deviation.

## Electronic supplementary material

Western blotting analysis of KDM8 expression in RWPE1 cells transfected with pcDNA-KDM8 or control empty vector (EV)

Western blotting analysis of KDM8 knockdown efficiency by shRNAs specifically targeting KDM8

Western blotting analysis of overexpression of KDM8 in LNCaP cells

Metabolic gene expressions in KDM8-overexpressed LNCaP cells (LNCaP-KDM8) and LNCaP-KDM8 cells with PKM2 knocked down by si-RNA targeting PKM2 (LNCaP-KDM8-si-PKM2)

Western blot analysis of PKM2 knockdown in LNCaP-KDM8 cells

qRT-PCR analysis of knockdown levels of genes in LNCaP-EV and LNCaP-KDM8 cells

EZH2 and ANCCA are critical for the growth of KDM8-overexpressing LNCaP cells

Xenografting experiments by using C4-2B and C4-2B-MDVR cell lines knocking down KDM8 with specific shRNA-KDM8 or control shRNA (LKO) in SCID mouse model

GSEA reveals biological pathways associated with KDM8 overexpression

Gleason Score of clinical prostate cancer tissues used in the study

ChIP qPCR primers used in the study

qPCR primers used in the study (Supplementary Information)

Antibodies used in this study

Figure Legends of Supplementary Information (ONC-2017-02309R)

## References

[1] Cui Y, Nadiminty N, Liu C, Lou W, Schwartz CT, Gao AC (2014). Upregulation of glucose metabolism by NF-kappaB2/p52 mediates enzalutamide resistance in castration-resistant prostate cancer cells. Endocr Relat Cancer.

[2] Shafi AA, Putluri V, Arnold JM, Tsouko E, Maity S, Roberts JM (2015). Differential regulation of metabolic pathways by androgen receptor (AR) and its constitutively active splice variant, AR-V7, in prostate cancer cells. Oncotarget.

[3] Choi SY, Xue H, Wu R, Fazli L, Lin D, Collins CC (2016). The MCT4 gene: a novel, potential target for therapy of advanced prostate cancer. Clin Cancer Res.

[4] Altenberg B, Greulich KO (2004). Genes of glycolysis are ubiquitously overexpressed in 24 cancer classes. Genomics.

[5] Ranasinghe WK, Xiao L, Kovac S, Chang M, Michiels C, Bolton D (2013). The role of hypoxia-inducible factor 1alpha in determining the properties of castrate-resistant prostate cancers. PLoS ONE.

[6] Yang W, Xia Y, Ji H, Zheng Y, Liang J, Huang W (2011). Nuclear PKM2 regulates beta-catenin transactivation upon EGFR activation. Nature.

[7] Wang HJ, Hsieh YJ, Cheng WC, Lin CP, Lin YS, Yang SF (2014). JMJD5 regulates PKM2 nuclear translocation and reprograms HIF-1alpha-mediated glucose metabolism. Proc Natl Acad Sci USA.

[8] Wong N, Yan J, Ojo D, De Melo J, Cutz JC, Tang D (2014). Changes in PKM2 associate with prostate cancer progression. Cancer Invest.

[9] Wang LY, Guo W, Kim K, Pochampalli M, Hung CL, Izumiya Y, et al. Histone demethylases in prostate cancer. In: Kumar R, editor. Nuclear signaling pathways and targeting transcription in cancer. New York, NY: Springer, 2013.

[10] Shin S, Janknecht R (2007). Diversity within the JMJD2 histone demethylase family. Biochem Biophys Res Commun.

[11] Wissmann M, Yin N, Muller JM, Greschik H, Fodor BD, Jenuwein T (2007). Cooperative demethylation by JMJD2C and LSD1 promotes androgen receptor-dependent gene expression. Nat Cell Biol.

[12] Kahl P, Gullotti L, Heukamp LC, Wolf S, Friedrichs N, Vorreuther R (2006). Androgen receptor coactivators lysine-specific histone demethylase 1 and four and a half LIM domain protein 2 predict risk of prostate cancer recurrence. Cancer Res.

[13] Cloos PA, Christensen J, Agger K, Maiolica A, Rappsilber J, Antal T (2006). The putative oncogene GASC1 demethylates tri- and dimethylated lysine 9 on histone H3. Nature.

[14] Chu CH, Wang LY, Hsu KC, Chen CC, Cheng HH, Wang SM (2014). KDM4B as a target for prostate cancer: structural analysis and selective inhibition by a novel inhibitor. J Med Chem.

[15] Duan L, Rai G, Roggero C, Zhang QJ, Wei Q, Ma SH (2015). KDM4/JMJD2 histone demethylase inhibitors block prostate tumor growth by suppressing the expression of AR and BMYB-regulated genes. Chem Biol.

[16] Oh S, Janknecht R (2012). Histone demethylase JMJD5 is essential for embryonic development. Biochem Biophys Res Commun.

[17] Hsia DA, Tepper CG, Pochampalli MR, Hsia EY, Izumiya C, Huerta SB (2010). KDM8, a H3K36me2 histone demethylase that acts in the cyclin A1 coding region to regulate cancer cell proliferation. Proc Natl Acad Sci USA.

[18] Zhu H, Hu S, Baker J (2014). JMJD5 regulates cell cycle and pluripotency in human embryonic stem cells. Stem Cells.

[19] Zhao Z, Sun C, Li F, Han J, Li X, Song Z (2015). Overexpression of histone demethylase JMJD5 promotes metastasis and indicates a poor prognosis in breast cancer. Int J Clin Exp Pathol.

[20] Zhang R, Huang Q, Li Y, Song Y, Li Y (2015). JMJD5 is a potential oncogene for colon carcinogenesis. Int J Clin Exp Pathol.

[21] Ishimura A, Minehata K, Terashima M, Kondoh G, Hara T, Suzuki T (2012). Jmjd5, an H3K36me2 histone demethylase, modulates embryonic cell proliferation through the regulation of Cdkn1a expression. Development.

[22] Huang X, Zhang S, Qi H, Wang Z, Chen HW, Shao J (2015). JMJD5 interacts with p53 and negatively regulates p53 function in control of cell cycle and proliferation. Biochim Biophys Acta.

[23] Marcon E, Ni Z, Pu S, Turinsky AL, Trimble SS, Olsen JB (2014). Human-chromatin-related protein interactions identify a demethylase complex required for chromosome segregation. Cell Rep.

[24] Amendola PG, Zaghet N, Ramalho JJ, Vilstrup Johansen J, Boxem M, Salcini AE (2017). JMJD-5/KDM8 regulates H3K36me2 and is required for late steps of homologous recombination and genome integrity. PLoS Genet.

[25] Wu J, He Z, Yang XM, Li KL, Wang DL, Sun FL. RCCD1 depletion attenuates TGF-beta-induced EMT and cell migration by stabilizing cytoskeletal microtubules in NSCLC cells. Cancer Lett. 2017;400:18–29. 10.1016/j.canlet.2017.04.021 28455245

[26] Youn MY, Yokoyama A, Fujiyama-Nakamura S, Ohtake F, Minehata K, Yasuda H (2012). JMJD5, a Jumonji C (JmjC) domain-containing protein, negatively regulates osteoclastogenesis by facilitating NFATc1 protein degradation. J Biol Chem.

[27] Liu H, Wang C, Lee S, Deng Y, Wither M, Oh S (2017). Clipping of arginine-methylated histone tails by JMJD5 and JMJD7. Proc Natl Acad Sci USA.

[28] Shen J, Xiang X, Chen L, Wang H, Wu L, Sun Y, et al. JMJD5 cleaves monomethylated histone H3 N-tail under DNA damaging stress. EMBO Rep. 2017;18:2131–43. 10.15252/embr.201743892 PMC5709736 28982940

[29] Ishimura A, Terashima M, Tange S, Suzuki T (2016). Jmjd5 functions as a regulator of p53 signaling during mouse embryogenesis. Cell Tissue Res.

[30] Wu BH, Chen H, Cai CM, Fang JZ, Wu CC, Huang LY (2016). Epigenetic silencing of JMJD5 promotes the proliferation of hepatocellular carcinoma cells by down-regulating the transcription of CDKN1A 686. Oncotarget.

[31] He Z, Wu J, Su X, Zhang Y, Pan L, Wei H (2016). JMJD5 (Jumonji Domain-containing 5) associates with spindle microtubules and is required for proper mitosis. J Biol Chem.

[32] Huang X, Zhang L, Qi H, Shao J, Shen J (2013). Identification and functional implication of nuclear localization signals in the N-terminal domain of JMJD5. Biochimie.

[33] Velasco AM, Gillis KA, Li Y, Brown EL, Sadler TM, Achilleos M (2004). Identification and validation of novel androgen-regulated genes in prostate cancer. Endocrinology.

[34] DePrimo SE, Diehn M, Nelson JB, Reiter RE, Matese J, Fero M, et al. Transcriptional programs activated by exposure of human prostate cancer cells to androgen. Genome Biol. 2002;3:RESEARCH0032. 10.1186/gb-2002-3-7-research0032 PMC126237 12184806

[35] Duan Z, Zou JX, Yang P, Wang Y, Borowsky AD, Gao AC (2013). Developmental and androgenic regulation of chromatin regulators EZH2 and ANCCA/ATAD2 in the prostate Via MLL histone methylase complex. Prostate.

[36] Liu C, Lou W, Zhu Y, Yang JC, Nadiminty N, Gaikwad NW (2015). Intracrine Androgens and AKR1C3 Activation Confer Resistance to Enzalutamide in Prostate Cancer. Cancer Res.

[37] Ku SY, Rosario S, Wang Y, Mu P, Seshadri M, Goodrich ZW (2017). Rb1 and Trp53 cooperate to suppress prostate cancer lineage plasticity, metastasis, and antiandrogen resistance. Science.

[38] Luo W, Semenza GL (2012). Emerging roles of PKM2 in cell metabolism and cancer progression. Trends Endocrinol Metab.

[39] Chaneton B, Hillmann P, Zheng L, Martin AC, Maddocks OD, Chokkathukalam A (2012). Serine is a natural ligand and allosteric activator of pyruvate kinase M2. Nature.

[40] Keller KE, Tan IS, Lee YS (2012). SAICAR stimulates pyruvate kinase isoform M2 and promotes cancer cell survival in glucose-limited conditions. Science.

[41] Hitosugi T, Kang S, Vander Heiden MG, Chung TW, Elf S, Lythgoe K (2009). Tyrosine phosphorylation inhibits PKM2 to promote the Warburg effect and tumor growth. Sci Signal.

[42] Rush J, Moritz A, Lee KA, Guo A, Goss VL, Spek EJ (2005). Immunoaffinity profiling of tyrosine phosphorylation in cancer cells. Nat Biotechnol.

[43] Lv L, Li D, Zhao D, Lin R, Chu Y, Zhang H (2011). Acetylation targets the M2 isoform of pyruvate kinase for degradation through chaperone-mediated autophagy and promotes tumor growth. Mol Cell.

[44] Anastasiou D, Poulogiannis G, Asara JM, Boxer MB, Jiang JK, Shen M (2011). Inhibition of pyruvate kinase M2 by reactive oxygen species contributes to cellular antioxidant responses. Science.

[45] Luo W, Hu H, Chang R, Zhong J, Knabel M, O’Meally R (2011). Pyruvate kinase M2 is a PHD3-stimulated coactivator for hypoxia-inducible factor 1. Cell.

[46] Gao X, Wang H, Yang JJ, Liu X, Liu ZR (2012). Pyruvate kinase M2 regulates gene transcription by acting as a protein kinase. Mol Cell.

[47] Yang W, Zheng Y, Xia Y, Ji H, Chen X, Guo F (2012). ERK1/2-dependent phosphorylation and nuclear translocation of PKM2 promotes the Warburg effect. Nat Cell Biol.

[48] Varambally S, Dhanasekaran SM, Zhou M, Barrette TR, Kumar-Sinha C, Sanda MG (2002). The polycomb group protein EZH2 is involved in progression of prostate cancer. Nature.

[49] Yang YA, Yu J (2013). EZH2, an epigenetic driver of prostate cancer. Protein Cell.

[50] Cao Q, Yu J, Dhanasekaran SM, Kim JH, Mani RS, Tomlins SA (2008). Repression of E-cadherin by the polycomb group protein EZH2 in cancer. Oncogene.

[51] Shin YJ, Kim JH (2012). The role of EZH2 in the regulation of the activity of matrix metalloproteinases in prostate cancer cells. PLoS ONE.

[52] Zhang Q, Padi SK, Tindall DJ, Guo B (2014). Polycomb protein EZH2 suppresses apoptosis by silencing the proapoptotic miR-31. Cell Death Dis.

[53] Beltran H, Rickman DS, Park K, Chae SS, Sboner A, MacDonald TY (2011). Molecular characterization of neuroendocrine prostate cancer and identification of new drug targets. Cancer Discov.

[54] Chng KR, Chang CW, Tan SK, Yang C, Hong SZ, Sng NY (2012). A transcriptional repressor co-regulatory network governing androgen response in prostate cancers. EMBO J.

[55] Xu K, Wu ZJ, Groner AC, He HH, Cai C, Lis RT (2012). EZH2 oncogenic activity in castration-resistant prostate cancer cells is Polycomb-independent. Science.

[56] Alumkal JJ, Herman JG (2012). Distinct epigenetic mechanisms distinguish TMPRSS2-ERG fusion-positive and -negative prostate cancers. Cancer Discov.

[57] Clermont PL, Lin D, Crea F, Wu R, Xue H, Wang Y (2015). Polycomb-mediated silencing in neuroendocrine prostate cancer. Clin Epigenetics.

[58] Liu C, Lou W, Zhu Y, Nadiminty N, Schwartz CT, Evans CP (2014). Niclosamide inhibits androgen receptor variants expression and overcomes enzalutamide resistance in castration-resistant prostate cancer. Clin Cancer Res.

[59] Li H, Hassona MD, Lack NA, Axerio-Cilies P, Leblanc E, Tavassoli P (2013). Characterization of a new class of androgen receptor antagonists with potential therapeutic application in advanced prostate cancer. Mol Cancer Ther.

[60] Ma AH, Xia L, Desai SJ, Boucher DL, Guan Y, Shih HM (2006). Male germ cell-associated kinase, a male-specific kinase regulated by androgen, is a coactivator of androgen receptor in prostate cancer cells. Cancer Res.

[61] Zou JX, Guo L, Revenko AS, Tepper CG, Gemo AT, Kung HJ (2009). Androgen-induced coactivator ANCCA mediates specific androgen receptor signaling in prostate cancer. Cancer Res.

[62] Yang P, Guo L, Duan ZJ, Tepper CG, Xue L, Chen X (2012). Histone methyltransferase NSD2/MMSET mediates constitutive NF-kappaB signaling for cancer cell proliferation, survival, and tumor growth via a feed-forward loop. Mol Cell Biol.

[63] Rhodes DR, Yu J, Shanker K, Deshpande N, Varambally R, Ghosh D (2004). ONCOMINE: a cancer microarray database and integrated data-mining platform. Neoplasia.

