# Supplementary figures and images for: KDM8/JMJD5 as a dual coactivator of AR and PKM2 integrates AR/EZH2 network and tumor metabolism in CRPC
Source: Oncogene. 2018 Aug 2;38(1):17–32. doi: 10.1038/s41388-018-0414-x (PMC6755995; doi:10.1038/s41388-018-0414-x)

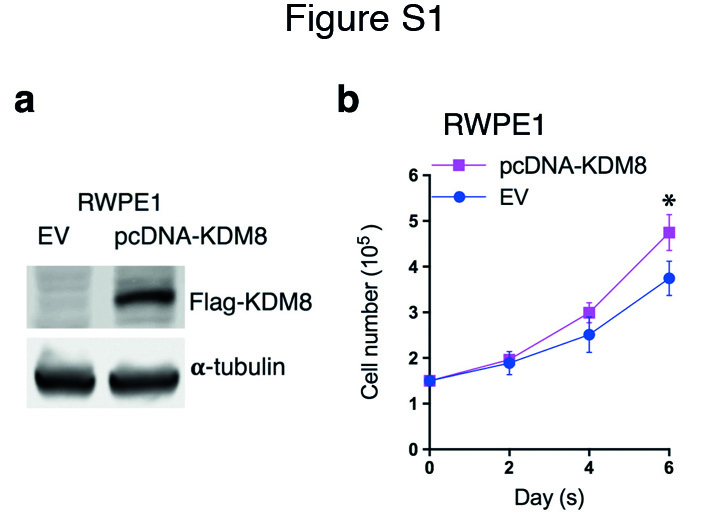

Supplement: Supplementary file 1 — Western blotting analysis of KDM8 expression in RWPE1 cells transfected with pcDNA-KDM8 or control empty vector (EV) [file 41388_2018_414_MOESM1_ESM.jpg]

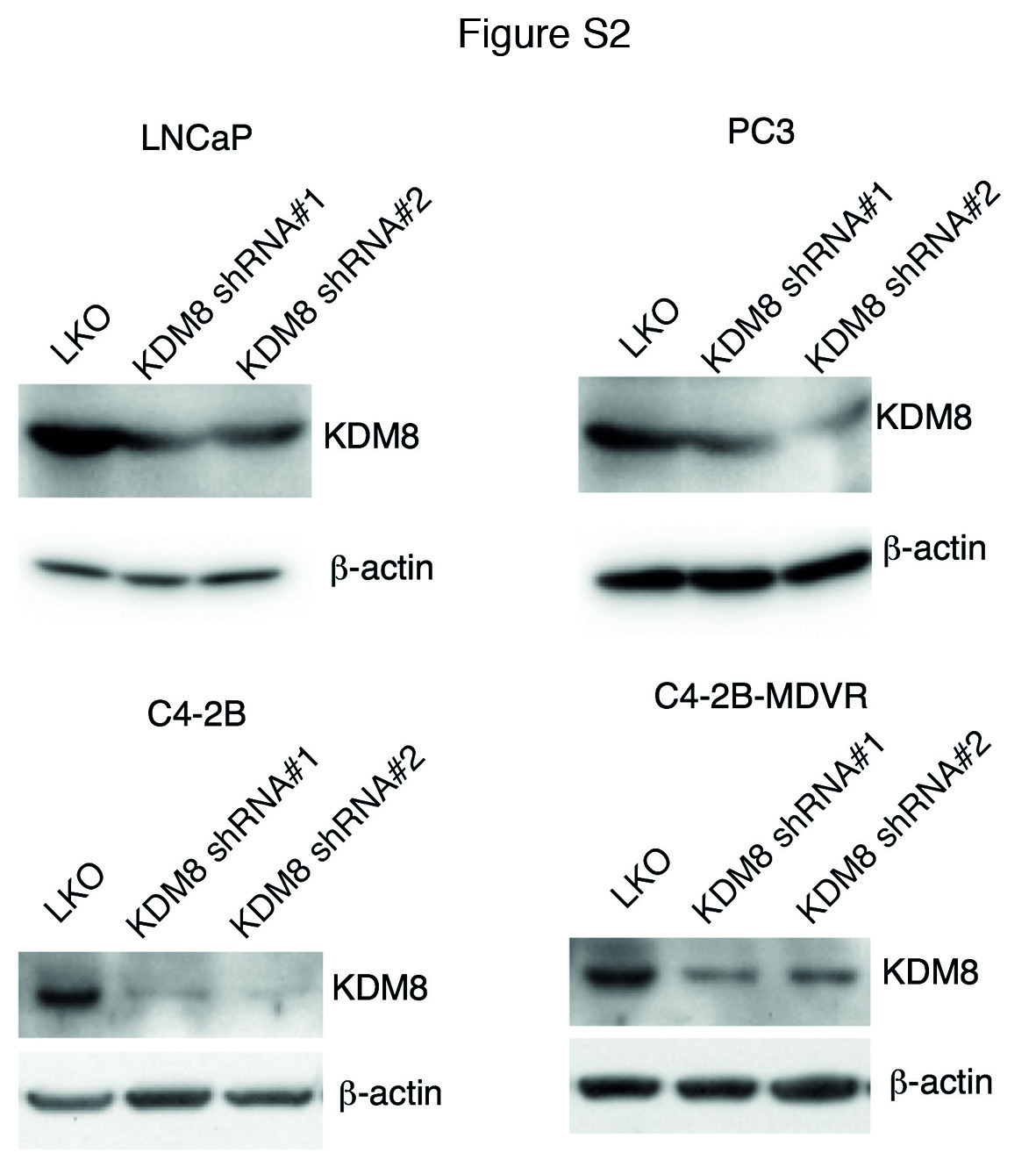

Supplement: Supplementary file 2 — Western blotting analysis of KDM8 knockdown efficiency by shRNAs specifically targeting KDM8 [file 41388_2018_414_MOESM2_ESM.jpg]

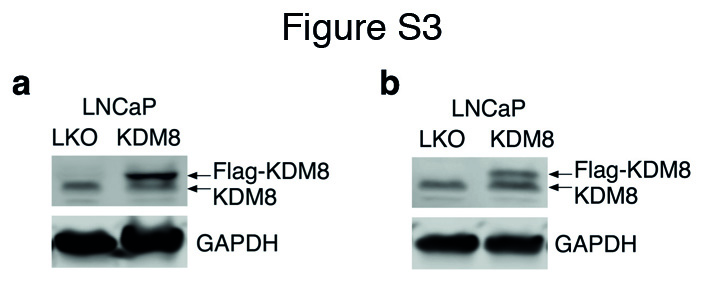

Supplement: Supplementary file 3 — Western blotting analysis of overexpression of KDM8 in LNCaP cells [file 41388_2018_414_MOESM3_ESM.jpg]

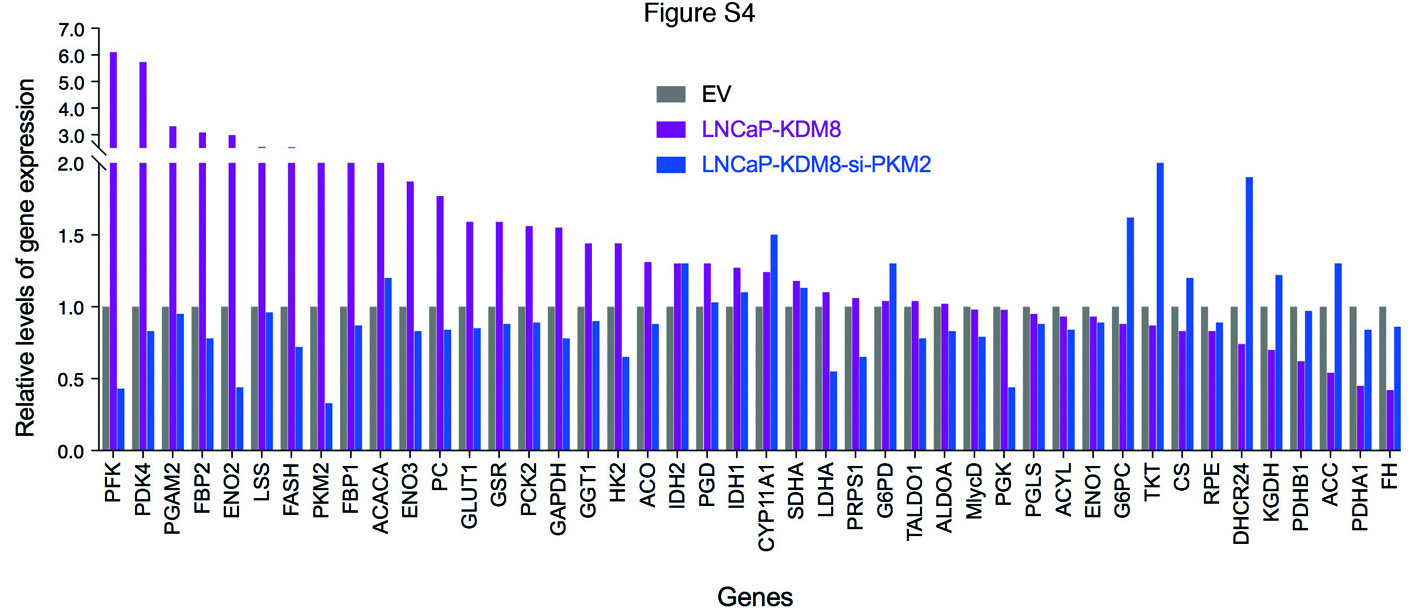

Supplement: Supplementary file 4 — Metabolic gene expressions in KDM8-overexpressed LNCaP cells (LNCaP-KDM8) and LNCaP-KDM8 cells with PKM2 knocked down by si-RNA targeting PKM2 (LNCaP-KDM8-si-PKM2) [file 41388_2018_414_MOESM4_ESM.jpg]

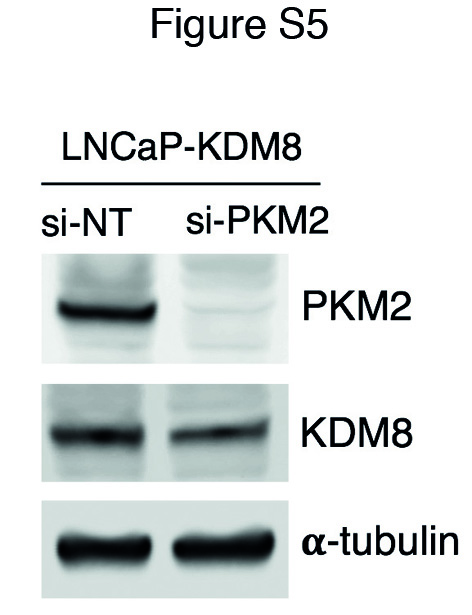

Supplement: Supplementary file 5 — Western blot analysis of PKM2 knockdown in LNCaP-KDM8 cells [file 41388_2018_414_MOESM5_ESM.jpg]

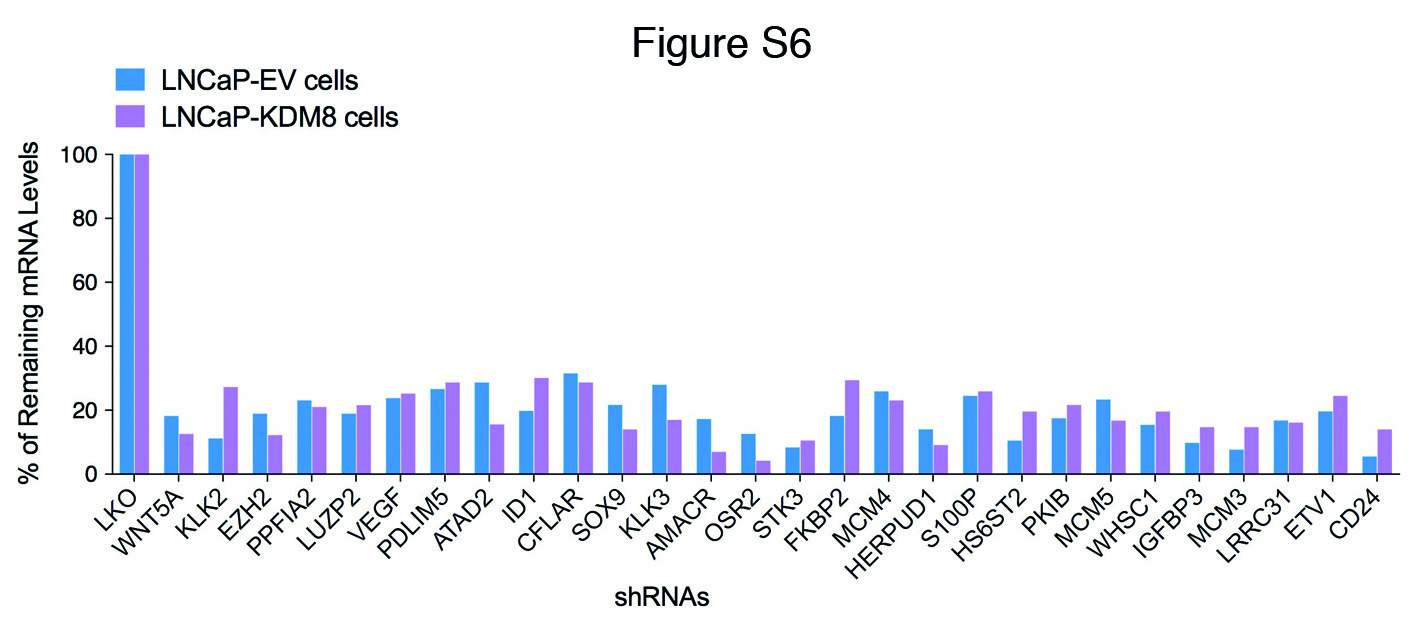

Supplement: Supplementary file 6 — qRT-PCR analysis of knockdown levels of genes in LNCaP-EV and LNCaP-KDM8 cells [file 41388_2018_414_MOESM6_ESM.jpg]

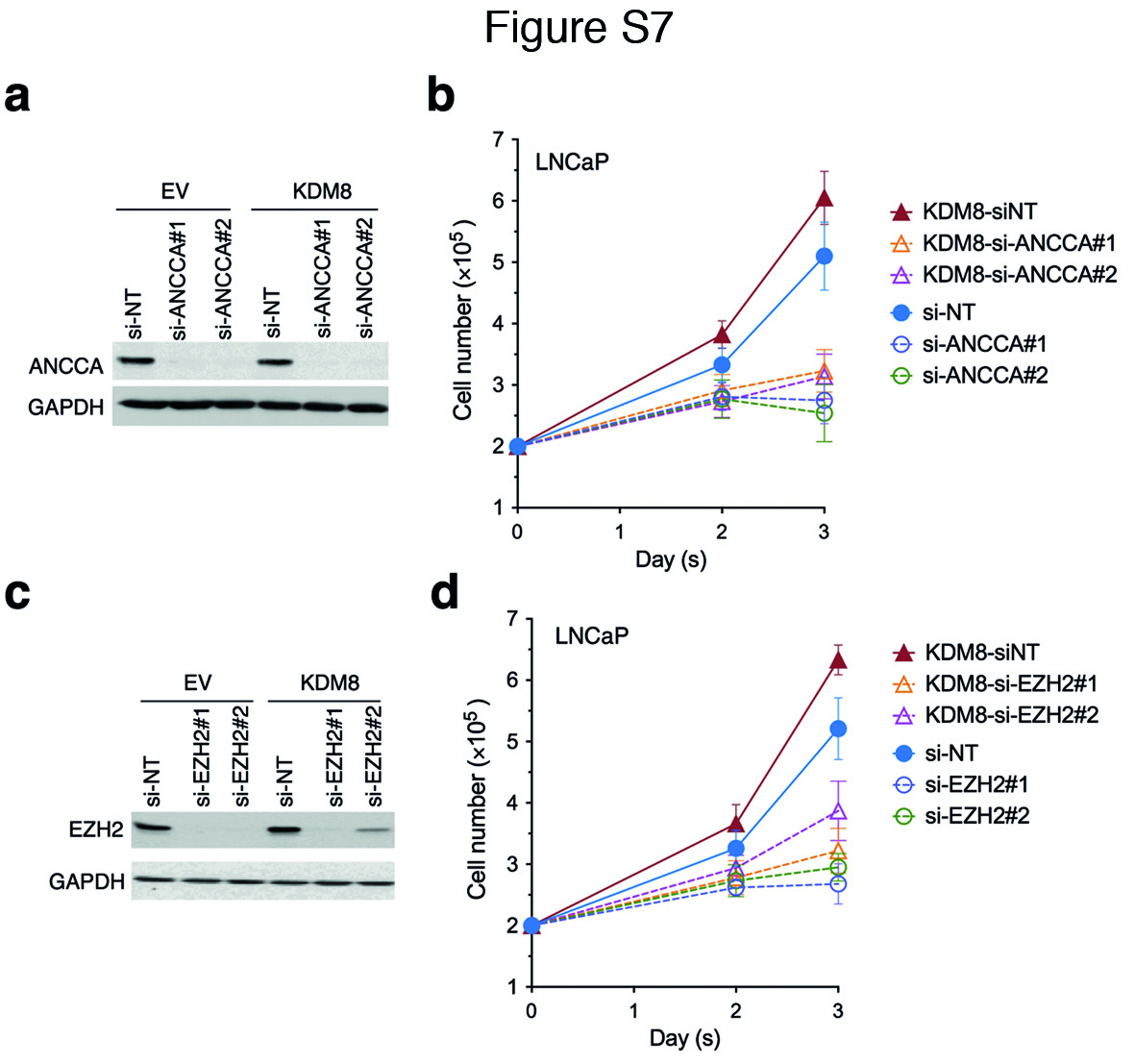

Supplement: Supplementary file 7 — EZH2 and ANCCA are critical for the growth of KDM8-overexpressing LNCaP cells [file 41388_2018_414_MOESM7_ESM.jpg]

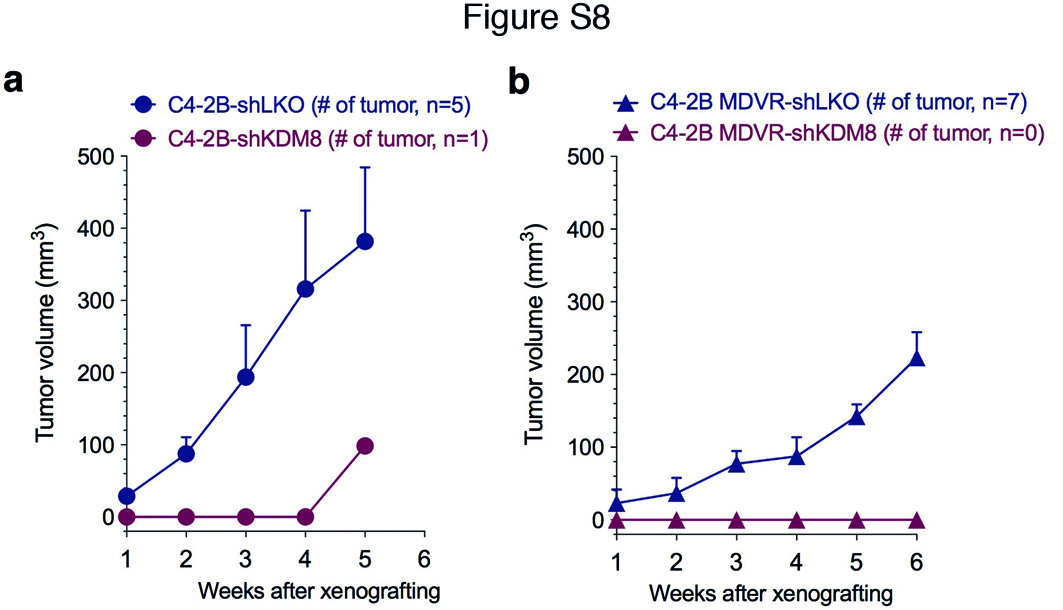

Supplement: Supplementary file 8 — Xenografting experiments by using C4-2B and C4-2B-MDVR cell lines knocking down KDM8 with specific shRNA-KDM8 or control shRNA (LKO) in SCID mouse model [file 41388_2018_414_MOESM8_ESM.jpg]

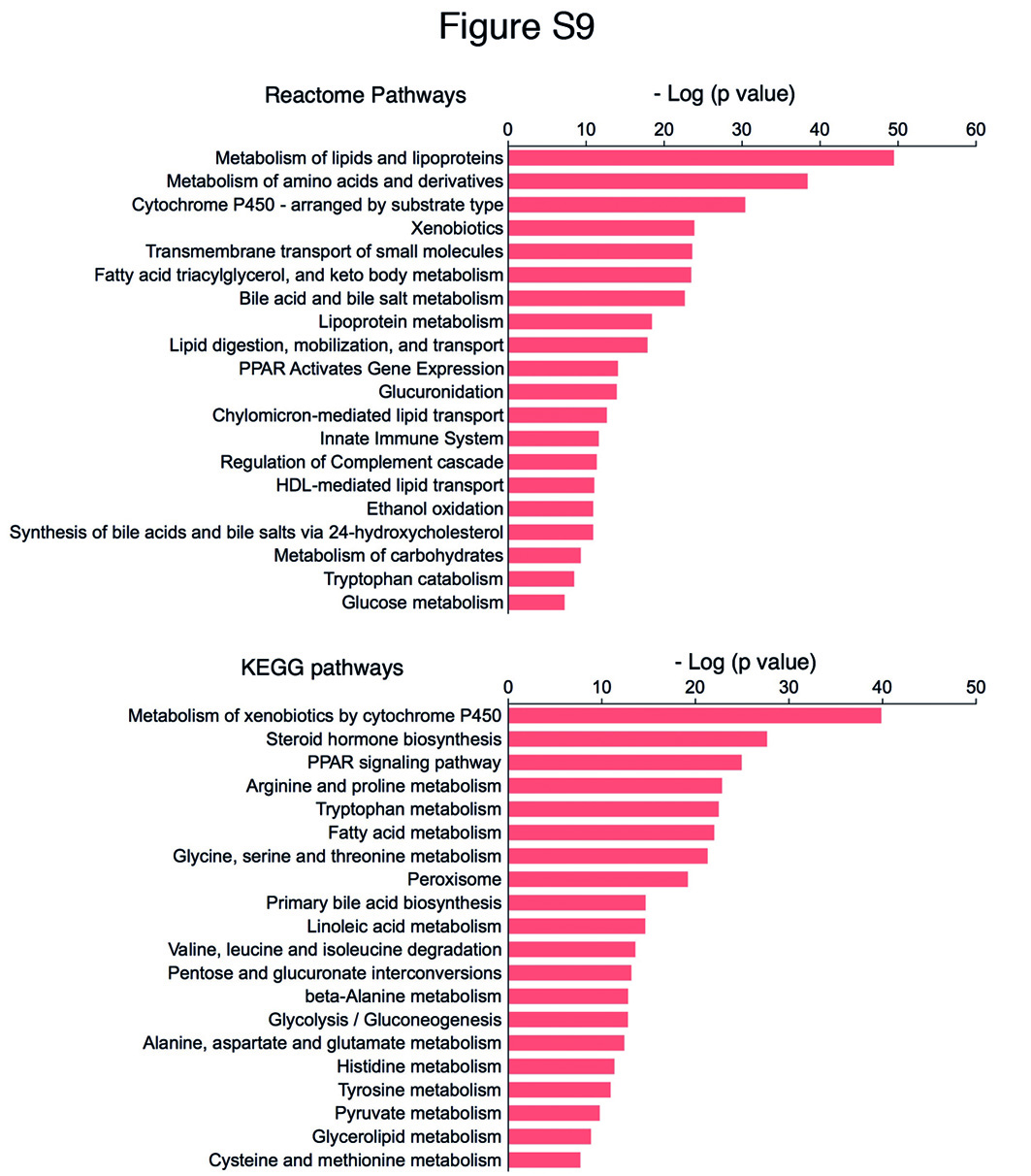

Supplement: Supplementary file 9 — GSEA reveals biological pathways associated with KDM8 overexpression [file 41388_2018_414_MOESM9_ESM.jpg]
