# Supplementary material for: KDM8/JMJD5 as a dual coactivator of AR and PKM2 integrates AR/EZH2 network and tumor metabolism in CRPC
Source: Oncogene. 2018 Aug 2;38(1):17–32. doi: 10.1038/s41388-018-0414-x (PMC6755995; doi:10.1038/s41388-018-0414-x)
Supplement: Supplementary file 10 — Gleason Score of clinical prostate cancer tissues used in the study [file 41388_2018_414_MOESM10_ESM.pdf]

PR952 (total 48 cases, 40 adenocarcinomas)

	KDM8 IHC Score			
	0 - 1	2 - 3	total	*p* value*
Adenocarcinomas				
Gleason score 2-6:	2	1	3	
7-10:	8	27	35	0.164
Normal adjacent to tumor:	7	1	8	

PR751 (total 73 scorable cases, 63 adenocarcinomas)

	KDM8 IHC Score			
	0 - 1	2 - 3	total	*p* value**
Adenocarcinomas				
Gleason score 2-6:	27	8	35	
7-10:	7	21	28	<0.001
Hyperplasia:	6	4	10	

* by Fisher's exact test; ** by chi-square test.
