# Supplementary material for: KDM8/JMJD5 as a dual coactivator of AR and PKM2 integrates AR/EZH2 network and tumor metabolism in CRPC
Source: Oncogene. 2018 Aug 2;38(1):17–32. doi: 10.1038/s41388-018-0414-x (PMC6755995; doi:10.1038/s41388-018-0414-x)
Supplement: Supplementary file 11 — ChIP qPCR primers used in the study [file 41388_2018_414_MOESM11_ESM.docx]

**Table S2. ChIP qPCR primers used in the study**

PSA-ARE III (enhancer):

PSA-F4 5’ GGGGTTTGTGCCACTGGTGAG3’

PSA-R4 5’GGGAGGCAATTCTCCATGGTT3’

PSA-ARE II/I (promoter):

PSA-F3 5’GCCAAGACATCTATTTCAGGAGC3’

PSA-R3 5’CCCACACCCAGAGCTGTGGAAGG3’

ANCCA/ATAD2-Enhancer

F12 5’GCAAGCACCATGAAGCCTGGC3’

R7 5’CCAGGTGTGGTGGTTCATGTC3’

ANCCA/ATAD2 promoter

pF1: 5’TAGAACAGCAGGCTCGAAACT

pR1: 5’CAAAATTCCAAACGGGCTACG

EZH2 Promoter

3F 5’CAGCGAAAGAACAAAGAGACGGCG3’

3R 5’GACCGGACCGAGCGCCAAC3’
