# Supplementary material for: KDM8/JMJD5 as a dual coactivator of AR and PKM2 integrates AR/EZH2 network and tumor metabolism in CRPC
Source: Oncogene. 2018 Aug 2;38(1):17–32. doi: 10.1038/s41388-018-0414-x (PMC6755995; doi:10.1038/s41388-018-0414-x)
Supplement: Supplementary file 12 — qPCR primers used in the study (Supplementary Information) [file 41388_2018_414_MOESM12_ESM.docx]

**qPCR primers used in this study (Supplementary Information)**

WNT5A-F: 5'-GATGGCTGGAAGTGCAATGT-3'

WNT5A-R: 5'-ACCTAGCGACCACCAAGAAT-3'

KLK2-F: 5'-TTTCTGGATGCTGGACACCT-3'

KLK2-R: 5'-TCAGTAGGACTCAGGAGGCT-3'

EZH2-F: 5'-CCACAGTGTTACCAGCATTTG-3'

EZH2-R: 5'-ACTGTTATTGGGAAGCCGTC-3'

PPFIA2-F: 5'-AGCGACTGAGGGTTTCTTTAG-3'

PPFIA2-R: 5'-GATGTTCTGACTCTGTGGATCC-3'

LUZP2-F: 5'-AGCTCTTGACAGGGAGTCAC-3'

LUZP2-R: 5'-GAGGAGGCTGATCACGGAAA-3'

VEGF-F: 5'-CTTCTGAGTTGCCCAGGAGA-3'

VEGF-R: 5'-CTGTCATGGGCTGCTTCTTC-3'

PDLIM5-F: TGCCAGCAAGAAGAGACTGA-3'

PDLIM5-R: AGCGAGACTGAGTTGTTCCA-3'

ATAD2-F: 5'-ATCTTCCGCAGGACCAAGAA-3'

ATAD2-R: 5'-TTTCCTCCGCCTCTCAAAGT-3'

ID1-F: 5'-GGTGAGCAAGGTGGAGATTC-3'

ID1-R: 5'-GGATTCCGAGTTCAGCTCCA-3'

CFLAR-F: 5'-GCTCTACAGAGTGAGGCGAT-3'

CFLAR-R: 5'-CCAATCTCTGCCATCAGCAC-3'

SOX9-F: 5'-GCCCGATCTGAAGAAGGAGA-3'

SOX9-R: 5'-CAGTCGTAGCCTTTGAGCAC-3'

KLK3-F: 5'-GAGTGACATGTGCTGGACAC-3'

KLK3-R: 5'-CTTGCTGTGAGTGTCTGGTG-3'

AMACR-F: 5'-GCTAGTGCTGGACCTGAAG-3'

AMACR-R: 5'-AGCCTTGGATTTTCCCGC-3'

OSR2-F: 5'-GCAAGAGGATCCGCCTAAGA-3'

OSR2-R: 5'-TTTACTGAGGCCCGAGATGG-3'

STK3-F: 5'-AGTGGCATTACCATCTGCCT-3'

STK3-R: 5'-TCTTCTCCTTGTGTGCTCCA-3'

FKBP2-F: 5'-GTCCTGCACATGCACTACAC-3'

FKBP2-R: 5'-AAGACAAAGGGCTGGTTCTG-3'

MCM4-F: 5'-TTCTTTGACCGTTACCCTGAC-3'

MCM4-R: 5-GGGATGTCCTGATCACCATG-3'

HERPUD1-F: 5'-GGCCACCGTTGTTATGTACC-3'

HERPUD1-R: 5'-TCAGGATCAGTGCCTTCCTG-3'

S100P-F: 5'-CAAGGTGCTGATGGAGAAGG-3'

S100P-R: 5'-ACGAACACGATGAACTCACTG-3'

HS6ST2-F: 5'-CGAGCCAAAGTGGAGTGATG-3'

HS6ST2-R: 5'-GAGCCACTTCACAAGCAGAG-3'

PKIB-F: 5'-GACACGGCTGTCTTCTTTCC-3'

PKIB-R: 5'-CGGTCAGATCCACATTAGCC-3'

MCM5-F: 5'-TGACTTCATGCCCACCATCT-3'

MCM5-R: 5'-GCATCACATCCCTCTCCTCA-3'

WHSC1-F: 5'-CCTCCAACAGCATCATCTGC-3'

WHSC1-R: 5'-CGTCAGGCATCTCGATGTTC-3'

IGFBP3-F: 5'-CAACTGTGGCCATGACTGAG-3'

IGFBP3-R: 5'-CAGACCTTCTTGGGTTTGGC-3'

MCM3-F: 5'-AGCGACGTTATTCTGATCTCAC-3'

MCM3-R: 5'-GATGGTGATGGTCTGGTGATC-3'

LRRC31-F: 5'-TCTTCAAGTGCTGAGGCTGA-3'

LRRC31-R: 5'-GACCCGTCTGTATGACCGAT-3'

ETV1-F: 5'-GGGAAGGACCCACATACCAA-3'

ETV1-R: 5'-CTCGACCAGTCCAGGCAATA-3'

CD24-F: 5'-CCAACTAATGCCACCACCA-3'

CD24-R: 5'- ACGAAGAGACTGGCTGTTGA-3'
