# Supplementary material for: KDM8/JMJD5 as a dual coactivator of AR and PKM2 integrates AR/EZH2 network and tumor metabolism in CRPC
Source: Oncogene. 2018 Aug 2;38(1):17–32. doi: 10.1038/s41388-018-0414-x (PMC6755995; doi:10.1038/s41388-018-0414-x)
Supplement: Supplementary file 13 — Antibodies used in this study [file 41388_2018_414_MOESM13_ESM.docx]

**Table S3. Antibodies used in this study**

**Antibody: Vendor: Catalog Number:**

Anti-AR Millipore 06-680

Anti-KDM8 Homemade

Anti-Flag tag Sigma F1804

Anti-Myc tag Cell Signaling 2276S

Anti-GAPDH Santa Cruz sc-365062

Anti-PKM2 Cell Signaling 4053S

Anti-Histone H3 Cell Signaling 4499S

Anti-EZH2 Active Motif 39934

Anti-ANCCA Abcam Ab118664

Anti-alpha-tubulin Santa Cruz sc-5286

Anti-bata-actin Cell Signaling 4967S
