# Supplementary material for: KDM8/JMJD5 as a dual coactivator of AR and PKM2 integrates AR/EZH2 network and tumor metabolism in CRPC
Source: Oncogene. 2018 Aug 2;38(1):17–32. doi: 10.1038/s41388-018-0414-x (PMC6755995; doi:10.1038/s41388-018-0414-x)
Supplement: Supplementary file 14 — Figure Legends of Supplementary Information (ONC-2017-02309R) [file 41388_2018_414_MOESM14_ESM.docx]

**Figure Legends of Supplementary Information (ONC-2017-02309R)**

**Figure legends:**

**Figure S1**. Western blotting analysis of KDM8 expression in RWPE1 cells transfected with pcDNA-KDM8 or control empty vector (EV). α-tubulin was used as an internal control. (**b**) Cell proliferation analysis of RWPE1 cells overexpressing KDM8. Cells were transfected with KDM8-expressing or EV control vectors same as (**a**). Live cells were stained with trypan blue (0.4%) and counted at 0, 2, 4, and 6 days post-transfection. *p-value* was obtained by *Student’s t* test.

**Figure S2.** Western blotting analysis of KDM8 knockdown efficiency by shRNAs specifically targeting KDM8 (KDM8 shRNA#1 and KDM8 shRNA#2). Prostate cancer cell lines as indicated were infected with lentivirus carrying shRNAs or an empty vector (LKO). The infected cells were selected with antibiotics and subjected to western blotting with anti-KDM8 and β-actin. β-actin served as internal controls. These are western blot controls for Figure 2a.

**Figure S3**. Western blotting analysis of overexpression of KDM8 in LNCaP cells. Cells were infected with KDM8-overexpressing vector (marked as KDM8) and control (LKO) lentivirus as indicated. Cell lysates were prepared and subjected to western blotting with anti-KDM8 and anti-GAPDH antibodies. GAPDH served as internal controls. These are western blot controls for Figure 2b (**a**) and Figure 2c (**b**).

**Figure S4**. Metabolic gene expressions in KDM8-overexpressed LNCaP cells (LNCaP-KDM8) and LNCaP-KDM8 cells with PKM2 knocked down by si-RNA targeting PKM2 (LNCaP-KDM8-si-PKM2). The gene expression levels were measured by qRT-PCR. Data were analyzed as relative fold change as compared to the EV control or si-NT after normalizing to internal control, 16S rRNA.

**Figure S5**. Western blot analysis of PKM2 knockdown in LNCaP-KDM8 cells. α-tubulin was served as an internal control.

**Figure S6**. qRT-PCR analysis of knockdown levels of genes in LNCaP-EV and LNCaP-KDM8 cells. The expression levels of the genes were normalized to18S rRNA and expressed as percentage of remaining mRNA levels normalized to the control (shRNA-LKO).

**Figure S7**. EZH2 and ANCCA are critical for the growth of KDM8-overexpressing LNCaP cells (marked as KDM8). LNCaP-EV (marked as EV) was used as a control. Two siRNAs of si-EZH2 and si-ANCCA were used for each gene knockdown. (**a, c**) Western blots of EZH2 and ANCCA showing the efficiency of knockdown. (**b, d**) The growths of the two cell lines treated with or without siRNAs are indicated.

**Figure S8**. Xenografting experiments by using C4-2B and C4-2B-MDVR cell lines knocking down KDM8 with specific shRNA-KDM8 or control shRNA (LKO) in SCID mouse model. The mice were grouped randomly. Five mice each group were used. Each mouse was intraperitoneally injected with the lentivirus-transducted cells in cell numbers of 1.0 × 10^6^ mixed with Matrigel as described in Experimental Procedures. Tumor volumes were measured once a week.

**Figure S9**. GSEA reveals biological pathways associated with KDM8 overexpression.

**Table S1.** Gleason Score of clinical prostate cancer tissues used in the study.

**Table S2.** ChIP qPCR primers used in the study.

**Table S3.** qPCR primers used in the study (Supplementary Information).

**Table S4.** Antibodies used in this study.
